# Systematic analysis of associations between obesity and memory decline

**DOI:** 10.1007/s11357-025-01725-3

**Published:** 2025-06-09

**Authors:** Ying Yue Huang, Wei Sen Zhang, Jiao Wang, Chao Qiang Jiang, Feng Zhu, Ya Li Jin, Kar Keung Cheng, Tai Hing Lam, Lin Xu

**Affiliations:** 1 School of Public Health, Sun Yat-Sen University, 74 Zhongshan 2nd Road, Guangzhou, Guangdong Province China; 2 Guangzhou Twelfth People’s Hospital, Guangzhou, 510620 China; 3 Department of Applied Health Sciences, School of Health Sciences, College of Medicine and Health, University of Birmingham, Birmingham, UK; 4 School of Public Health, The University of Hong Kong, Hong Kong, China; 5 Greater Bay Area Public Health Research Collaboration, Greater Bay Area, China

**Keywords:** Obesity, Memory function, Memory decline, Mendelian randomisation

## Abstract

Despite the development of numerous obesity indicators, the optimal measure associated with memory remains to be elucidated. We examined and compared these associations whilst also exploring potential interactions with education. We analysed the associations between 20 obesity indicators and memory (measured by Delayed Word Recall Test (DWRT)) in middle-aged and older participants from Guangzhou Biobank Cohort Study by linear regression and stratification analyses. We used two-sample Mendelian randomisation (2SMR) to analyse the associations of obesity with cognitive performance. Of 20 obesity indicators, 6 and 7 were associated with poorer memory function measured by baseline and follow-up DWRT; 7 and 10 with faster memory decline measured by mean annual change (MAC) and MAC rate of DWRT. Central obesity measured by waist-to-hip-to-height ratio (WHHR) *z*-score showed the greatest effect size, with the *β*s (95% CIs) being − 0.09 (− 0.11 to − 0.07), − 0.07 (− 0.09 to − 0.04), − 0.02 (− 0.03 to − 0.01) and − 0.49 (− 0.69 to − 0.29), respectively. The associations were weaker in those with higher education (*P* for education-interaction from 0.008 to 0.049). In 2SMR, body mass index (BMI), waist circumference and BMI-adjusted waist-to-hip ratio were negatively associated with cognitive performance using inverse-variance weighted method, with *β*s (95% CIs) being − 0.11 (− 0.15 to − 0.07), − 0.07 (− 0.12 to − 0.02) and − 0.06 (− 0.09 to − 0.02), respectively. In conclusion, obesity, especially central obesity measured by WHHR, was associated with poorer memory function and faster memory decline in middle-aged and older people, with a weaker association observed amongst those with higher education.

**Graphical Abstract:**

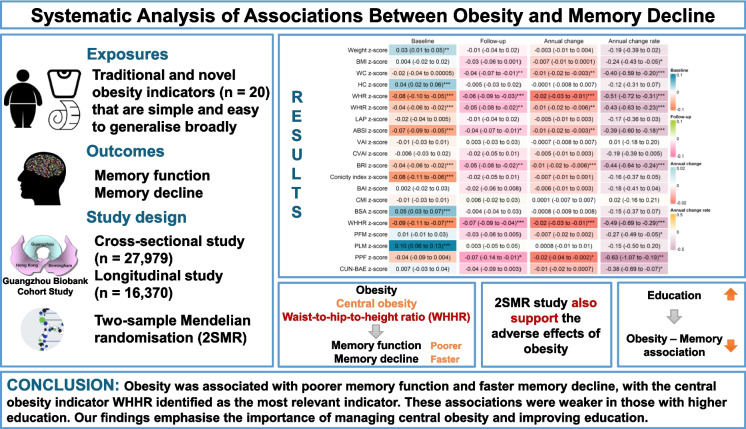

**Supplementary Information:**

The online version contains supplementary material available at 10.1007/s11357-025-01725-3.

## Introduction

The global increase in life expectancy has led to a substantial rise in the prevalence of dementia [1]. Cognitive decline, particularly in memory function, is an early symptom of dementia, guiding early intervention strategies to prevent memory decline in the ageing population.

The 2024 report of the Lancet standing Commission suggests that nearly half of dementias may be prevented or delayed with attention to 14 modifiable risk factors, and one of them is obesity [2]. Body mass index (BMI) is the most used obesity indicator, but the results on its association with dementia risk appeared to be conflicting [3–5], probably due to its limitation to grossly estimate adiposity distribution and metabolic health [6]. Therefore, incorporating additional factors such as body shape and other features is necessary to more precisely determine individual risk of obesity-related conditions [6]. Several new obesity indicators have been proposed to complement BMI, such as lipid accumulation product (LAP) and a body shape index (ABSI). However, previous studies examining the association between obesity and memory still predominantly relied on traditional obesity indicators, such as BMI, waist circumference (WC) and waist-to-hip ratio (WHR) [7, 8]. No study has systematically compared the predictive power of traditional and novel obesity indicators in determining late-life memory function and memory decline.

The interaction of obesity and education on memory has been explored by only a limited number of studies. One western cross-sectional study showed that there were no effects of education or BMI on working memory [9]. Conversely, another western cross-sectional study showed that individuals with higher BMI, categorised as overweight or obese, exhibited poorer nonverbal memory performance exclusively amongst less highly educated individuals [10]. Furthermore, a western cohort study demonstrated a negative association between middle-age BMI and late-life cognition, whilst the effect of BMI was attenuated in individuals with higher education [11]. Only one Chinese longitudinal study explored and found that contrary to expectations, higher visceral adiposity index (VAI) levels were associated with improved episodic memory, and there was no education-interaction [12]. The moderating effect of education on the association between obesity and memory remains inconclusive, with no study exploring obesity indicators beyond BMI and VAI. Therefore, it remains to further examine and confirm the effect modification of education.

Furthermore, Mendelian randomisation (MR) has been used for making causal inferences from observational data [13]. Our literature search up to May 2025 yielded seven MR studies on the associations of obesity with cognitive function [14–20]. Several studies consistently reported negative causal associations of obesity indicators with cognitive function, including BMI [16–20], WC [16, 20], WHR [15, 16, 18], visceral adipose tissue (VAT) [16] and body fat percentage (BF%) [18]. However, two studies reported no effect of BMI on cognitive function [14, 15], and one study reported no effects of WHR and BF% on cognitive function [14]. With the advent of constantly updated genome-wide association studies (GWAS) with larger sample sizes, we conducted an updated MR analysis to reassess the effects of BMI, WC, WHR and VAT on cognitive function. Moreover, only two studies have employed both observational study and MR to investigate the association between obesity and cognitive function [16, 17]. However, one study was limited by its cross-sectional design, which restricted its extrapolation on cognitive decline [16], whilst the other assessed obesity using BMI alone, which restricted a comprehensive evaluation of obesity [17].

Hence, using Guangzhou Biobank Cohort Study (GBCS) data, we analysed and compared the associations of 20 obesity indicators with memory function and memory decline in middle-aged and older participants and analysed potential interactions of obesity indicators with education. Moreover, we conducted an updated two-sample MR study for potential causal associations of obesity indicators with cognitive performance.

## Methods

### Conventional observational study

#### Study participants

The GBCS is a three-way collaboration amongst the Guangzhou Twelfth People’s Hospital and the Universities of Hong Kong, China and Birmingham, UK. At baseline from 2003 to 2008, 30,430 participants aged 50 years or above were recruited. All surviving participants were invited for the first follow-up physical examination in March 2008 to December 2012. Details of baseline examination and some results from the follow-up examination have been reported previously [21–24].

Briefly, recruitment of GBCS participants was from a community social and welfare association, the Guangzhou Health and Happiness Association for the Respective Elders (GHHARE). GHHARE is unofficially aligned with the municipal government and has ten branches throughout all districts of Guangzhou. Membership of GHHARE is open to Guangzhou permanent residents aged 50 years or above with a nominal fee of 4 CNY (about 50 US cents) per month. About 7% of local residents in this age group are enrolled in the GHHARE, and 33% of them were included in GBCS. The baseline and the first follow-up examinations included a face-to-face interview by trained nurses using a computer-assisted standardised questionnaire that included demographic characteristics and lifestyle factors and assessment of anthropometric parameters and lipids. The study was approved by the Guangzhou Medical Ethics Committee of the Chinese Medical Association, and all participants provided written informed consent prior to participation.

#### Exposures

After a systematic search, we identified 20 obesity indicators which could be analysed in our study, including weight, BMI, WC, hip circumference (HC), WHR, waist-to-height ratio (WHtR), LAP, ABSI, VAI, Chinese VAI (CVAI), body roundness index (BRI), conicity index, body adiposity index (BAI), cardiometabolic index (CMI), body surface area (BSA), waist-to-hip-to-height ratio (WHHR), predicted fat mass (PFM), predicted lean mass (PLM), predicated percent fat (PPF) and Clínica Universidad de Navarra-Body Adiposity Estimator (CUN-BAE). Details of the search process and measurements of obesity indicators have been shown in the Supplementary methods. For comparison, obesity indicators were standardised using *z*-score transformation for analysis of the associations with memory function.

#### Outcomes

Memory function was assessed by delayed 10-word recall test (DWRT) at both baseline (2003–2008) and follow-up (2008–2012) examinations as reported in previous GBCS papers [22, 25, 26]. Of the ten words, “arm”, “ticket”, “grass” and “letter” were retained from the original English language test [27]. “Book”, “stick”, “corner” and “stone” substituted “cabin”, “engine”, “pole” and “shore” as in the adapted Consortium 10-word list learning task [28]. To fit Chinese culture better, “soy sauce” and “chairman” replaced “butter” and “queen”. During the interview, the 10 words were read out to participants one by one, and then, they were asked to recall the words immediately. This procedure was repeated three times. After 5 min of answering other questions for distraction, participants were asked to recall as many words as they could remember. Participants were given one point for each correct word that they could recall, and the total number of correct words was recorded as DWRT score. Then, according to previous GBCS papers [25, 26], memory decline was calculated by mean annual change and mean annual rate of change in DWRT score. Mean annual change = (follow-up score – baseline score)/follow-up time, and mean annual rate of change = (mean annual change/baseline score) × 100. Memory impairment was defined by DWRT score < 4, corresponding to one standard deviation (SD) below the mean (mean ± SD: 5.5 ± 1.8).

### Mendelian randomisation

#### Genetic associations with exposures

To conduct an updated MR analysis, genetic associations with obesity indicators (BMI, WC, WHR adjusted for BMI (aWHR) and VAT) were obtained from the largest and most recent publicly available GWAS from Genetic Investigation of Anthropometric Traits (GIANT) or UK Biobank [29, 30]. Detailed information of each GWAS for the four obesity indicators is presented in Table S1.

#### Genetic associations with cognitive outcome

Cognitive performance was measured by the verbal-numerical reasoning score or by at least three neuropsychological tests or two IQ-test scores, with a higher score indicating better cognitive performance. Genetic associations with cognitive performance were also obtained from the largest and most recent publicly available GWAS from meta-analysis of the Cognitive Genomics Consortium (COGENT) and UK Biobank (*n* = 257,841) [31]. Table S1 summarises the detailed information for this GWAS.

### Statistical analysis

#### Conventional observational study

Chi-square test and analysis of variance were used to compare baseline characteristics of categorical and continuous variables according to presence of memory impairment. In cross-sectional analyses, multivariable linear regression was used to analyse the associations of obesity indicators with DWRT score at baseline. In longitudinal analyses, multivariable linear regression, Cox regression and generalised estimating equation (GEE) were used to analyse the associations of obesity indicators with follow-up DWRT score, DWRT change and the presence of new-onset memory impairment. For those with memory impairment at the follow-up examination, the censoring date was defined as the midpoint between the baseline and follow-up examinations. The results were presented as regression coefficients (*β*s), hazard ratios (HRs) and 95% confidence intervals (CIs). Potential confounders included sex, age (continuous), education (primary or low, secondary and college or above), occupation (manual, non-manual and others), personal income (< 10,000 RMB/year, 10,000–14,999 RMB/year, ≥ 15,000 RMB/year and not reported, US $1 ≈ 8 RMB), physical activity measured by International Physical Activity Questionnaire (IPAQ) (inactive, moderate and active) [32], alcohol drinking (never, former and current drinkers), smoking (never, former and current smokers), self-rated health (good and poor) and baseline DWRT score. Amongst them, definition of alcohol drinking was based on the usual frequency in the past 12 months, as described in our previous studies [33, 34]. Never drinkers were those who never consumed any alcoholic beverage during their life, former drinkers were those who had abstained from alcohol for at least 1 year, and current drinkers were those who ever drank in the past 12 months. Definition of smoking was based on two questions “do you smoke now?”, and “what is your past smoking habit?” as described in our previous studies [35–37]. Never smokers were those who did not use tobacco product during their life time, former smokers were those who used to smoke daily but had quitted smoking currently, and current smokers were those who has smoked at least one cigarette/day or seven cigarettes/week for at least half a year. We also analysed whether the associations varied by education (primary or less and secondary or more). Interactions were tested by fitting models with and without the interaction term, with statistical significance determined by the likelihood ratio test of the difference between the two models. Moreover, we used restricted cubic spline analysis to analyse the potential non-linear relationship between obesity and memory function at baseline or follow-up.

#### Mendelian randomisation

The causal associations of obesity with cognitive performance were analysed using two-sample MR. First, we obtained single nucleotide polymorphisms (SNPs) strongly (*P*-value < 5 × 10^−8^) associated with exposures. Second, linkage disequilibrium (LD) between SNPs was identified using “ld_clump” R package, and those highly correlated SNPs (*r*^2^ ≥ 0.001) with higher *P*-values were discarded. Third, we aligned the effect alleles of outcomes to be consistent with the effect alleles of exposures. Moreover, *F*-statistics of the instruments was calculated by the square of SNP-exposure association divided by its variance [38], and the mean *F*-statistics was used to assess instrument strength [39]. In the primary analyses, we used inverse-variance weighted (IVW) method. As sensitivity analyses, we repeated the analysis using weighted median estimator (WM), MR-Egger regression and MR Pleiotropy RESidual Sum and Outlier (MR-PRESSO). A zero intercept from MR-Egger (*P* > 0.05) indicates no potential horizontal pleiotropy.

All statistical analyses were done using Stata version 16.0 (StataCorp LP, College Station, TX) and R version 4.1.1 (R Foundation for Statistical Computing, Vienna, Austria). The “TwoSampleMR”, “MendelianRandomization” and “MRPRESSO” packages were used. All tests were two-sided, with *P* < 0.05 as statistically significant.

## Results

### Characteristics of participants

Of 30,518 participants recruited from 2003 to 2008, after excluding those with duplicate information (*N* = 88) and missing information on obesity indicators (*N* = 303), DWRT score (*N* = 1230) and potential confounders (*N* = 1283), 27,979 participants with all variables of interest were included in cross-sectional analyses. During the first follow-up examination (2008–2012), 18,104 participants returned for repeated measurement. After excluding those with missing information on obesity indicators (*N* = 137), DWRT score in 2008–2012 (*N* = 517) and potential confounders (*N* = 1160), 16,370 participants were included in longitudinal analyses.

Table 1 shows that participants with baseline memory impairment were older, had higher proportion of men, those with lower education, manual occupation and lower personal income and current smokers (*P* from < 0.001 to 0.02), but lower proportion of those who were physically active, current alcohol users and with good health status (all *P* < 0.001). Moreover, amongst 20 obesity indicators, 12 were higher in participants with baseline memory impairment, including WC, WHR, WHtR, LAP, ABSI, CVAI, BRI, conicity index, BAI, CMI, WHHR and CUN-BAE, but PLM was lower (*P* from < 0.001 to 0.03). A similar pattern was seen in participants with follow-up memory impairment.

**Table 1:** Baseline characteristics of the study sample by baseline or follow-up memory impairment

	Cross-sectional data				Longitudinal data			
	Total	Baseline memory impairment		*P*-value	Total	Follow-up memory impairment		*P*-value
		No	Yes			No	Yes	
Number of participants (row percentage)	27,979 (100%)	24,316 (86.91)	3663 (13.09)		16,370 (100%)	14,957 (91.37)	1413 (8.63)	
Sex (%)								
Men	27.59	27.35	29.18	0.02	27.04	26.42	33.62	< 0.001
Women	72.41	72.65	70.82		72.96	73.58	66.38	
Age, years, mean (SD)	62.00 (7.07)	61.50 (6.93)	65.29 (7.13)	< 0.001	61.06 (6.76)	60.68 (6.63)	65.10 (6.76)	< 0.001
Education (%)								
Primary or below	42.85	39.37	65.98	< 0.001	38.64	36.31	63.27	< 0.001
Secondary	48.21	50.98	29.81		52.07	53.95	32.13	
College or above	8.94	9.65	4.20		9.29	9.73	4.60	
Occupation (%)								
Manual	60.99	59.58	70.38	< 0.001	59.93	58.92	70.63	< 0.001
Non-manual	24.00	25.14	16.38		24.82	25.52	17.41	
Others	15.01	15.28	13.24		15.25	15.56	11.96	
Personal income (%)								
< 10,000 RMB/year	33.45	31.81	44.39	< 0.001	32.00	31.11	41.40	< 0.001
10,000–14,999 RMB/year	43.28	44.18	37.29		44.84	45.38	39.14	
≥ 15,000 RMB/year	18.49	19.51	11.71		18.62	19.22	12.24	
Not reported	4.78	4.51	6.61		4.54	4.29	7.22	
Smoking status (%)								
Never	80.86	81.26	78.24	< 0.001	82.37	82.96	76.08	< 0.001
Former	9.12	8.98	10.07		8.38	8.06	11.75	
Current	10.02	9.77	11.68		9.25	8.97	12.17	
Alcohol drinking (%)								
Never	72.70	71.84	78.41	< 0.001	71.78	71.50	74.66	0.01
Former	3.50	3.43	3.90		3.37	3.34	3.68	
Current	23.81	24.73	17.69		24.86	25.16	21.66	
Physical activity (%)								
Inactive	8.02	8.02	8.08	< 0.001	8.12	8.23	6.94	0.01
Moderate	41.13	40.66	44.25		39.89	39.57	43.31	
Active	50.85	51.33	47.67		51.99	52.20	49.75	
Self-rated health (%)								
Poor	17.67	17.11	21.38	< 0.001	16.35	16.32	16.70	0.71
Good	82.33	82.89	78.62		83.65	83.68	83.30	
Weight, kg, mean (SD)	58.40 (9.62)	58.55 (9.59)	57.41 (9.76)	< 0.001	58.52 (9.51)	58.57 (9.48)	57.98 (9.75)	0.03
BMI, kg/m^2^, mean (SD)	23.78 (3.32)	23.79 (3.30)	23.73 (3.41)	0.29	23.78 (3.25)	23.77 (3.24)	23.78 (3.37)	0.95
WC, cm, mean (SD)	78.79 (8.98)	78.65 (8.94)	79.76 (9.18)	< 0.001	78.43 (8.82)	78.28 (8.78)	80.06 (9.06)	< 0.001
HC, cm, mean (SD)	90.74 (6.36)	90.80 (6.35)	90.36 (6.46)	< 0.001	90.74 (6.28)	90.76 (6.25)	90.59 (6.55)	0.36
WHR, mean (SD)	0.87 (0.07)	0.87 (0.07)	0.88 (0.07)	< 0.001	0.86 (0.07)	0.86 (0.07)	0.88 (0.07)	< 0.001
WHtR, mean (SD)	0.50 (0.06)	0.50 (0.06)	0.51 (0.06)	< 0.001	0.50 (0.06)	0.50 (0.06)	0.51 (0.06)	< 0.001
LAP,^a^ mean (SD)	34.64 (33.59)	34.29 (33.07)	36.99 (36.80)	< 0.001	33.75 (32.02)	33.61 (32.10)	35.16 (31.13)	0.09
ABSI, mean (SD)	0.08 (0.005)	0.076 (0.005)	0.078 (0.005)	< 0.001	0.08 (0.005)	0.076 (0.005)	0.078 (0.005)	< 0.001
VAI, mean (SD)	1.85 (1.85)	1.84 (1.85)	1.90 (1.86)	0.09	1.83 (1.82)	1.84 (1.85)	1.75 (1.40)	0.07
CVAI, mean (SD)	96.15 (41.35)	95.22 (41.10)	102.33 (42.45)	< 0.001	94.14 (40.53)	93.54 (40.32)	100.54 (42.16)	< 0.001
BRI, mean (SD)	3.49 (1.10)	3.46 (1.09)	3.69 (1.16)	< 0.001	3.43 (1.06)	3.41 (1.05)	3.69 (1.14)	< 0.001
Conicity index, mean (SD)	0.16 (0.01)	0.156 (0.01)	0.161 (0.02)	< 0.001	0.16 (0.01)	0.155 (0.01)	0.160 (0.01)	< 0.001
BAI, mean (SD)	28.46 (4.17)	28.41 (4.14)	28.80 (4.27)	< 0.001	28.38 (4.08)	28.35 (4.05)	28.65 (4.38)	0.009
CMI, mean (SD)	0.58 (0.60)	0.58 (0.60)	0.60 (0.61)	0.03	0.58 (0.59)	0.58 (0.60)	0.56 (0.45)	0.32
BSA, mean (SD)	1.58 (0.15)	1.58 (0.15)	1.55 (0.15)	< 0.001	1.58 (0.14)	1.58 (0.14)	1.57 (0.15)	0.002
WHHR, mean (SD)	0.006 (0.0005)	0.0055 (0.0005)	0.0057 (0.0005)	< 0.001	0.006 (0.0005)	0.0055 (0.0005)	0.0057 (0.0005)	< 0.001
PFM, mean (SD)	19.93 (6.22)	19.97 (6.19)	19.67 (6.40)	0.006	19.94 (6.10)	19.99 (6.06)	19.47 (6.51)	0.002
PLM, mean (SD)	37.21 (7.41)	37.31 (7.40)	36.50 (7.41)	< 0.001	37.31 (7.40)	37.32 (7.38)	37.24 (7.60)	0.70
PPF, mean (SD)	34.24 (7.22)	34.21 (7.18)	34.41 (7.48)	0.13	34.19 (7.12)	34.23 (7.06)	33.79 (7.79)	0.03
CUN-BAE, mean (SD)	33.43 (6.88)	33.40 (6.84)	33.66 (7.12)	0.03	33.36 (6.78)	33.38 (6.72)	33.16 (7.37)	0.25
DWRT score, mean (SD)	5.48 (1.84)	5.94 (1.47)	2.44 (0.88)	< 0.001	5.65 (1.80)	5.76 (1.77)	4.45 (1.68)	< 0.001

*ABSI* a body shape index, *BAI* body adiposity index, *BMI* body mass index, *BRI* body roundness index, *BSA* body surface area, *CMI* cardiometabolic index, *CUN-BAE* Clínica Universidad de Navarra-Body Adiposity Estimator, *CVAI* Chinese visceral adiposity index, *DWRT* delayed word recall test, *HC* hip circumference, *LAP* lipid accumulation product, *PFM* predicted fat mass, *PLM* predicted lean mass, *PPF* predicated percent fat, *SD* standard deviation, *VAI* visceral adiposity index, *WC* waist circumference, *WHHR* waist-to-hip-to-height ratio, *WHR* waist-to-hip ratio, *WHtR* waist-to-height ratio

^a^Only 27,650 participants with LAP greater than or equal to zero were included here

### Baseline obesity indicators and memory function at baseline

**Fig. 1:**
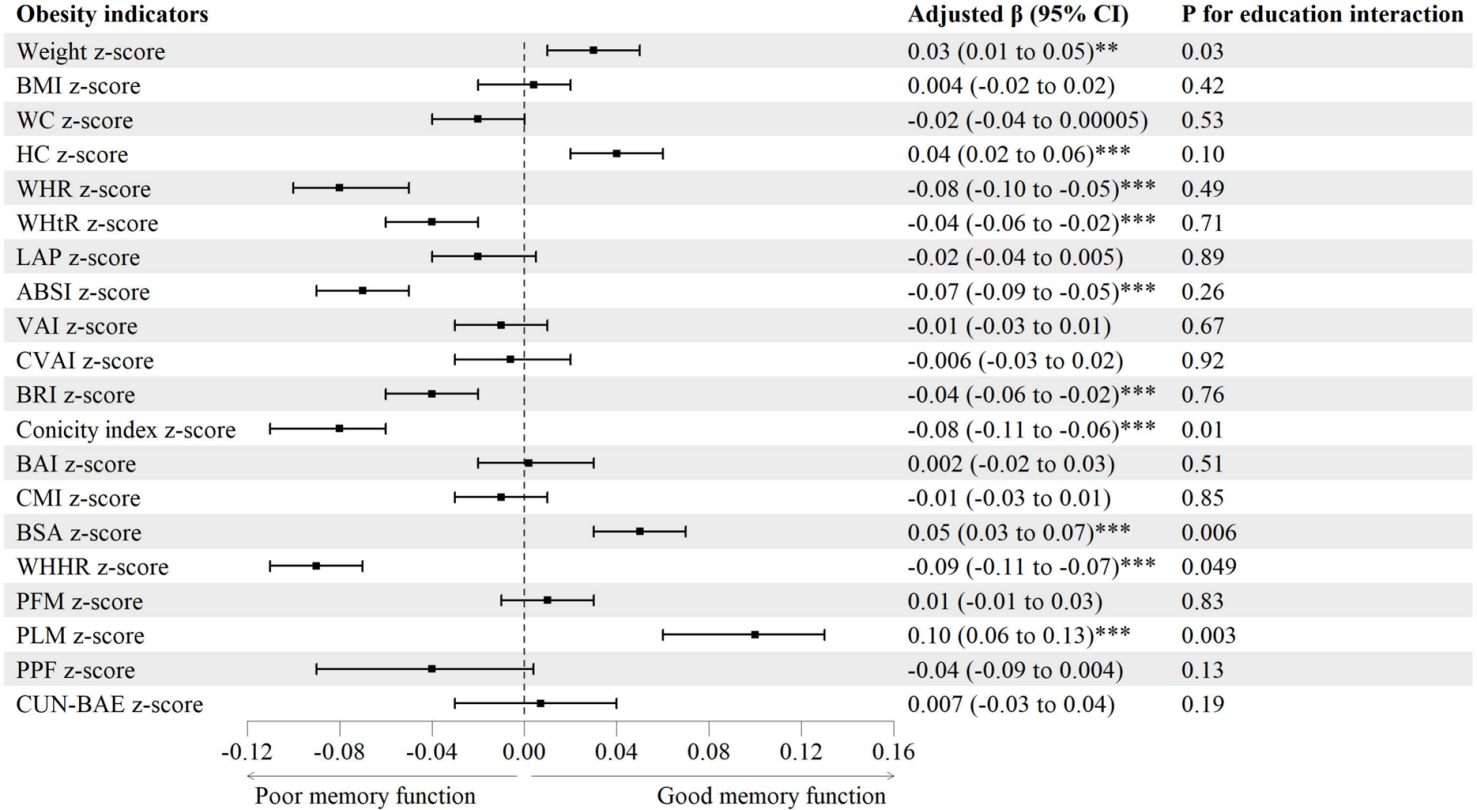
Associations of baseline obesity indicators with memory function at baseline. Note: ABSI, a body shape index; BAI, body adiposity index; BMI, body mass index; BRI, body roundness index; BSA, body surface area; CMI, cardiometabolic index; CUN-BAE, Clínica Universidad de Navarra-Body Adiposity Estimator; CVAI, Chinese visceral adiposity index; DWRT, delayed word recall test; HC, hip circumference; LAP, lipid accumulation product; PFM, predicted fat mass; PLM, predicted lean mass; PPF, predicated percent fat; Ref, reference; VAI, visceral adiposity index; WC, waist circumference; WHHR, waist-to-hip-to-height ratio; WHR, waist-to-hip ratio; WHtR, waist-to-height ratio. Adjusted *β* (95% CI): adjusted for sex, age, education, occupation, personal income, physical activity, drinking, smoking and self-rated health. *P < 0.05, **P < 0.01, ***P < 0.001

Figure 1 and Table S2 show that at baseline, after adjusting for sex, age, education, occupation, personal income, physical activity, drinking, smoking and self-rated health, WHR, WHtR, ABSI, BRI, conicity index and WHHR *z*-score were negatively associated with DWRT score, with adjusted *β*s (95% CIs) being − 0.08 (− 0.10 to − 0.05), − 0.04 (− 0.06 to − 0.02), − 0.07 (− 0.09 to − 0.05), − 0.04 (− 0.06 to − 0.02), − 0.08 (− 0.11 to − 0.06) and − 0.09 (− 0.11 to − 0.07), but those with higher weight, HC, BSA and PLM *z*-score showed higher DWRT score (*β*s (95% CIs): 0.03 (0.01 to 0.05), 0.04 (0.02 to 0.06), 0.05 (0.03 to 0.07) and 0.10 (0.06 to 0.13), respectively). Moreover, Fig. 1 and Table S3 show that at baseline, there were interactions between five obesity indicators *z*-score (weight, conicity index, BSA, WHHR and PLM) and education on DWRT score (*P* for interaction from 0.003 to 0.049). Subgroup analyses by education showed that the positive associations of weight, BSA and PLM *z*-score with DWRT score became weaker or even attenuated to null in those with higher education. The *β*s (95% CIs) of DWRT score for primary school or lower *versus* secondary school or higher were 0.05 (0.02 to 0.08) versus 0.02 (− 0.007 to 0.05), 0.08 (0.04 to 0.11) versus 0.04 (0.005 to 0.07) and 0.13 (0.07 to 0.19) versus 0.08 (0.04 to 0.13), respectively. Moreover, the negative associations of conicity index and WHHR *z*-score with DWRT score became weaker in those with higher education. The *β*s (95% CIs) were − 0.10 (− 0.11 to − 0.07) versus − 0.07 (− 0.10 to − 0.04) and − 0.11 (− 0.14 to − 0.08) versus − 0.08 (− 0.11 to − 0.05), respectively. Table S2 and Fig. S1 show non-linear negative associations of conicity index, WHHR and CUN-BAE with memory function at baseline.

### Baseline obesity indicators and memory function at follow-up

**Fig. 2:**
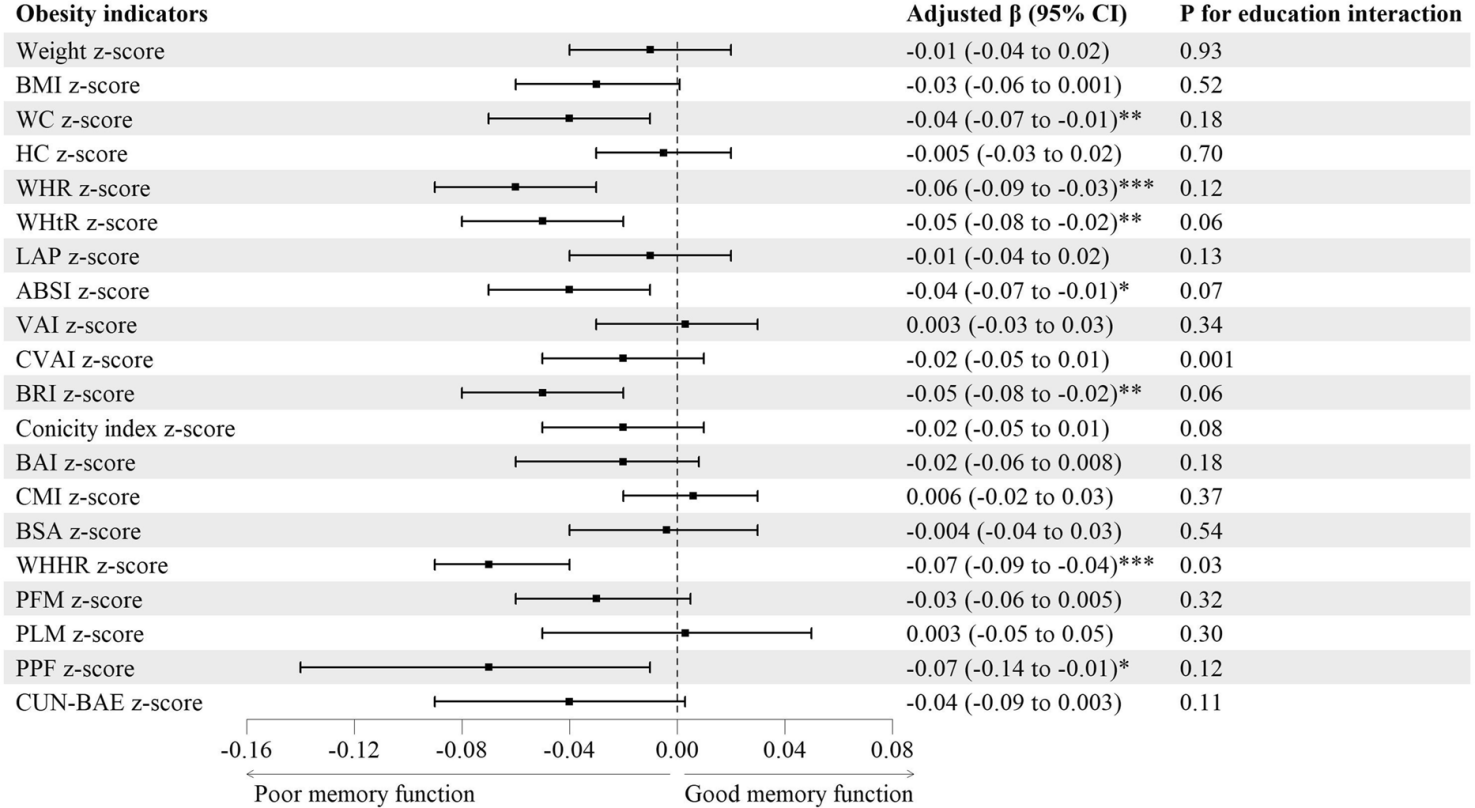
Associations of baseline obesity indicators with memory function at follow-up. Note: ABSI, a body shape index; BAI, body adiposity index; BMI, body mass index; BRI, body roundness index; BSA, body surface area; CMI, cardiometabolic index; CUN-BAE, Clínica Universidad de Navarra-Body Adiposity Estimator; CVAI, Chinese visceral adiposity index; DWRT, delayed word recall test; HC, hip circumference; LAP, lipid accumulation product; PFM, predicted fat mass; PLM, predicted lean mass; PPF, predicated percent fat; Ref, reference; VAI, visceral adiposity index; WC, waist circumference; WHHR, waist-to-hip-to-height ratio; WHR, waist-to-hip ratio; WHtR, waist-to-height ratio. Adjusted *β* (95% CI): adjusted for sex, age, education, occupation, personal income, physical activity, drinking, smoking, self-rated health and baseline DWRT score. *P < 0.05, **P < 0.01, ***P < 0.001

Figure 2 and Table S4 show that, after adjusting for confounders as above and baseline DWRT score, WC, WHR, WHtR, ABSI, BRI, WHHR and PPF *z*-score were negatively associated with follow-up DWRT score, with *β*s (95% CIs) being − 0.04 (− 0.07 to − 0.01), − 0.06 (− 0.09 to − 0.03), − 0.05 (− 0.08 to − 0.02), − 0.04 (− 0.07 to − 0.01), − 0.05 (− 0.08 to − 0.02), − 0.07 (− 0.09 to − 0.04) and − 0.07 (− 0.14 to − 0.01). Moreover, Fig. 2 and Table S5 show interactions between CVAI, WHHR *z*-score and education on follow-up DWRT score (*P* for interaction from 0.001 to 0.03). Subgroup analyses by education showed that higher CVAI *z*-score was associated with lower follow-up DWRT score only in those with lower education (*β* (95% CI) − 0.06 (− 0.11 to − 0.008)). Compared with participants with higher education, the association between WHHR *z*-score and follow-up DWRT score was stronger in those with lower education (*β* (95% CI) − 0.05 (− 0.09 to − 0.01) versus − 0.08 (− 0.13 to − 0.03)). Furthermore, higher obesity indicators *z*-score were also associated with higher odds of memory impairment at follow-up (Table S6), and the results were similar using GEE model (Table S7). Table S4 and Fig. S2 show non-linear negative associations of CVAI, WHHR and PPF with memory function at follow-up.

### Baseline obesity indicators and mean annual change of memory function

**Fig. 3:**
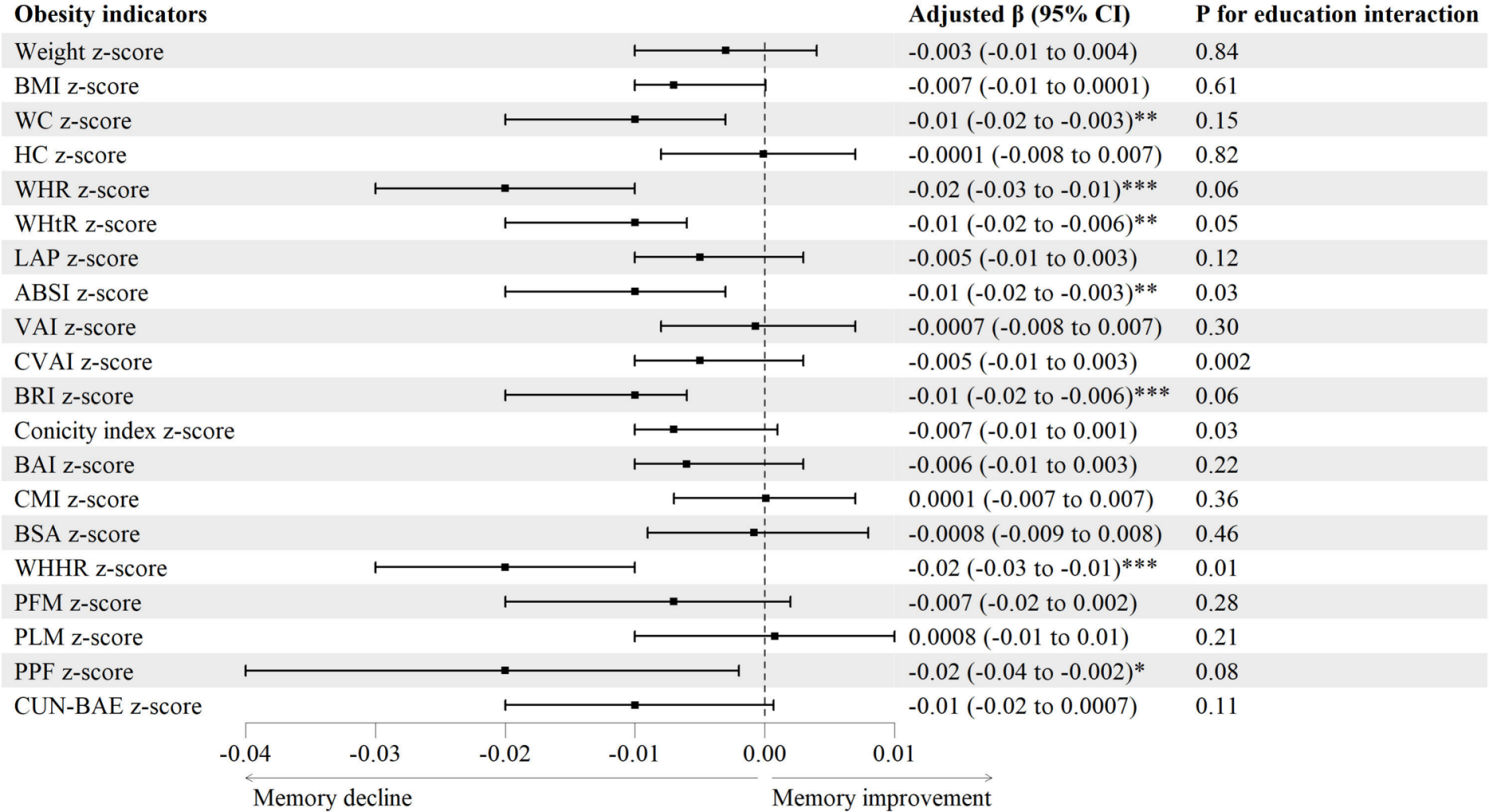
Associations of baseline obesity indicators with mean annual change of memory function. Note: ABSI, a body shape index; BAI, body adiposity index; BMI, body mass index; BRI, body roundness index; BSA, body surface area; CMI, cardiometabolic index; CUN-BAE, Clínica Universidad de Navarra-Body Adiposity Estimator; CVAI, Chinese visceral adiposity index; DWRT, delayed word recall test; HC, hip circumference; LAP, lipid accumulation product; PFM, predicted fat mass; PLM, predicted lean mass; PPF, predicated percent fat; Ref, reference; VAI, visceral adiposity index; WC, waist circumference; WHHR, waist-to-hip-to-height ratio; WHR, waist-to-hip ratio; WHtR, waist-to-height ratio. Adjusted *β* (95% CI): adjusted for sex, age, education, occupation, personal income, physical activity, drinking, smoking, self-rated health and baseline DWRT score. *P < 0.05, **P < 0.01, ***P < 0.001

Figure 3 and Table S8 show that after adjusting for confounders as above, higher WC, WHR, WHtR, ABSI, BRI, WHHR and PPF *z*-score were associated with greater decrease in mean annual change of DWRT score, with *β* (95% CI) being − 0.01 (− 0.02 to − 0.003), − 0.02 (− 0.03 to − 0.01), − 0.01 (− 0.02 to − 0.006), − 0.01 (− 0.02 to − 0.003), − 0.01 (− 0.02 to − 0.006), − 0.02 (− 0.03 to − 0.01) and − 0.02 (− 0.04 to − 0.002), respectively. Moreover, Fig. 3 and Table S9 show interactions between four obesity indicators (ABSI, CVAI, conicity index, WHHR *z*-score) and education on mean annual change of DWRT score (*P* for interaction from 0.002 to 0.03). Subgroup analyses by education showed that only in those with lower education, higher ABSI and CVAI *z*-score was associated with greater decrease in mean annual change of DWRT score (*β* (95% CI) − 0.02 (− 0.03 to − 0.003) and − 0.02 (− 0.03 to − 0.003), respectively). Compared with participants with higher education, the negative associations of conicity index and WHHR *z*-score with mean annual change of DWRT score were stronger in those with lower education (*β* (95% CI) − 0.005 (− 0.02 to 0.006) versus − 0.009 (− 0.02 to 0.004) and − 0.01 (− 0.02 to − 0.004) versus − 0.03 (− 0.04 to − 0.02), respectively).

### Baseline obesity indicators and mean annual change rate of memory function

**Fig. 4:**
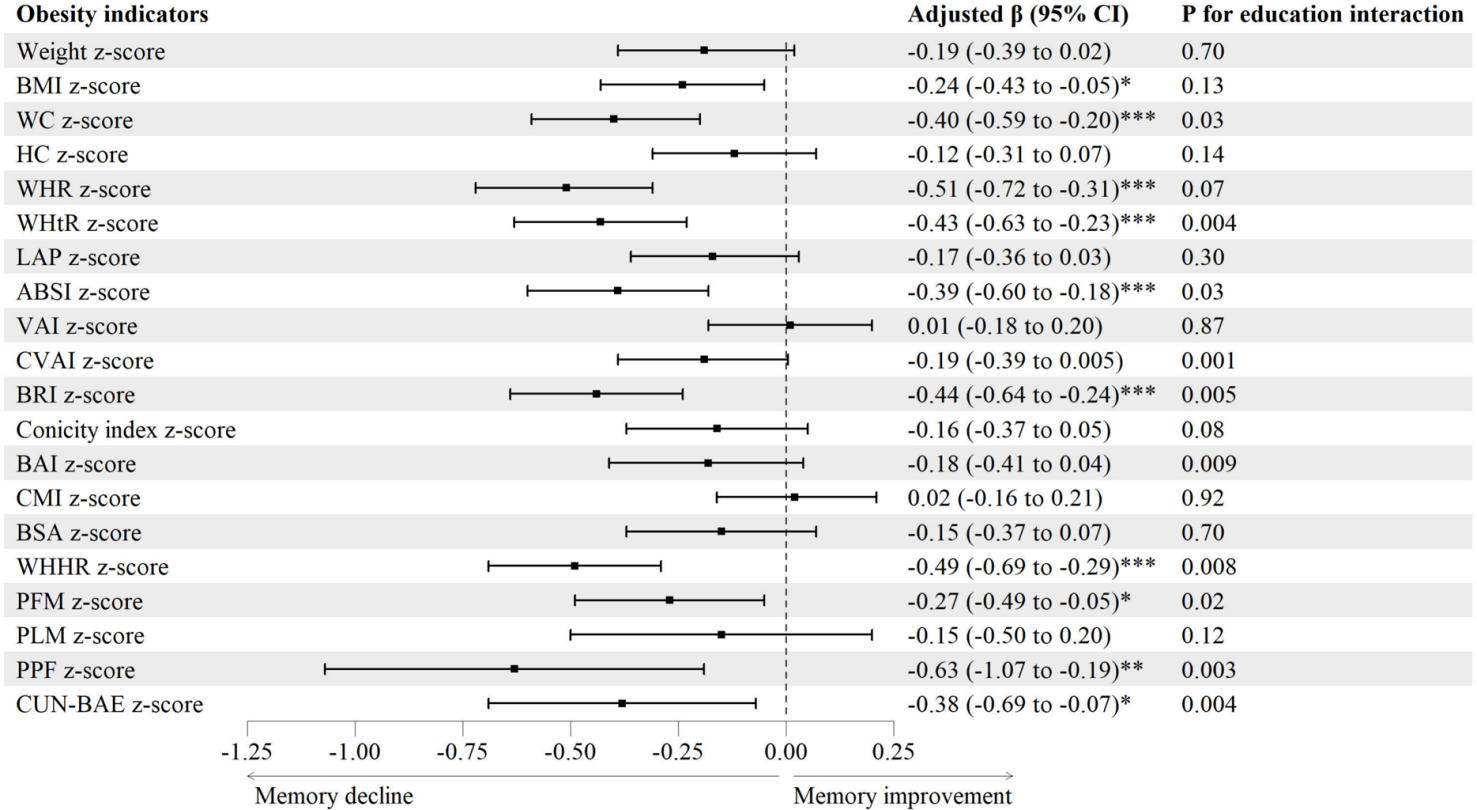
Associations of baseline obesity indicators with mean annual change rate of memory function. Note: ABSI, a body shape index; BAI, body adiposity index; BMI, body mass index; BRI, body roundness index; BSA, body surface area; CMI, cardiometabolic index; CUN-BAE, Clínica Universidad de Navarra-Body Adiposity Estimator; CVAI, Chinese visceral adiposity index; DWRT, delayed word recall test; HC, hip circumference; LAP, lipid accumulation product; PFM, predicted fat mass; PLM, predicted lean mass; PPF, predicated percent fat; Ref, reference; VAI, visceral adiposity index; WC, waist circumference; WHHR, waist-to-hip-to-height ratio; WHR, waist-to-hip ratio; WHtR, waist-to-height ratio. Adjusted *β* (95% CI): adjusted for sex, age, education, occupation, personal income, physical activity, drinking, smoking, self-rated health and baseline DWRT score. *P < 0.05, **P < 0.01, ***P < 0.001

Figure 4 and Table S10 show that after adjusting for confounders as above, higher BMI, WC, WHR, WHtR, ABSI, BRI, WHHR, PFM, PPF and CUN-BAE *z*-score were associated with greater decrease in mean annual change rate of DWRT score, with *β* (95% CI) being − 0.24 (− 0.43 to − 0.05), − 0.40 (− 0.59 to − 0.20), − 0.51 (− 0.72 to − 0.31), − 0.43 (− 0.63 to − 0.23), − 0.39 (− 0.60 to − 0.18), − 0.44 (− 0.64 to − 0.24), − 0.49 (− 0.69 to − 0.29), − 0.27 (− 0.49 to − 0.05), − 0.63 (− 1.07 to − 0.19) and − 0.38 (− 0.69 to − 0.07), respectively. Moreover, Fig. 4 and Table S11 show interactions between ten obesity indicators (WC, WHtR, ABSI, CVAI, BRI, BAI, WHHR, PFM, PPF and CUN-BAE) and education on mean annual change rate of DWRT score (*P* for interaction from 0.001 to 0.03). Subgroup analyses by education showed that the negative associations of these obesity indicators *z*-score with mean annual change rate of DWRT score became weaker in those with higher education.

### Mendelian randomisation

The number of SNPs associated with BMI, WC, aWHR and VAT at genome-wide significance (*P*-value < 5 × 10^−8^) and without high LD (*r*^2^ ≥ 0.001) was 516, 374, 307 and 5, respectively. Of these SNPs, 514, 370, 305 and 5 SNPs were found in outcome datasets, and subsequently, 10, 12, 13 and 0 SNPs were excluded due to being palindromic, respectively. Figure S3 shows the selection of genetic instruments for obesity indicators.

Table 2 shows negative associations of genetically determined level of BMI, WC and aWHR with cognitive performance using IVW, with *β* (95% CI) being − 0.11 (− 0.15 to − 0.07), − 0.07 (− 0.12 to − 0.02) and − 0.06 (− 0.09 to − 0.02), respectively. Similar results were found in the analyses using WM, MR Egger and MR-PRESSO. After removing overlapped SNPs between BMI and WC, the results were similar. Moreover, after removing one outlier SNP, the MR-PRESSO shows a marginally significant negative association between VAT and cognitive performance (− 0.17, 95% CI − 0.29 to − 0.04, *P* = 0.08). Similar trends were found in the analyses using IVW, WM and MR-Egger. The MR-Egger intercepts indicated no statistical evidence of horizontal pleiotropy (all *P* > 0.05).

**Table 2:** Associations of genetically determined obesity with cognitive performance

	Mendelian randomisation method	SNPs used	Mean *F*-statistic	*β*	95% confidence interval	*P*-value	Cochran’s *Q* (*I*^2^**)**	MR-Egger intercept (*P*-value)	Outliers from MR PRESSO
BMI	IVW	504	75	** − 0.11**	** − 0.15 to − 0.07**	** < 0.001**	2548.2 (80.3%)	− 0.001 (0.24)	rs10182181, rs10761785, rs10942267, rs11713193, rs11781699, rs11782074, rs12150665, rs13049280, rs13107325, rs13186194, rs13263601, rs1421334, rs1501673, rs17182027, rs17367750, rs215669, rs217433, rs2273175, rs2289379, rs273512, rs2984618, rs3101336, rs3754963, rs427943, rs4303732, rs4700608, rs6010784, rs6493498, rs6757852, rs7124681, rs738140, rs7498665, rs768023, rs889398, rs9320823, rs9937053, rs9944219
	WM			** − 0.09**	** − 0.12 to − 0.05**	** < 0.001**			
	MR Egger			− 0.06	− 0.16 to 0.05	0.28			
	MR-PRESSO			** − 0.10**	** − 0.13 to − 0.07**	** < 0.001**			
WC	IVW	358	58	** − 0.07**	** − 0.12 to − 0.02**	**0.005**	1885.2 (81.1%)	− 0.001 (0.24)	rs11787216, rs1191600, rs12103006, rs215669, rs2253310, rs2306593, rs2470946, rs2568958, rs2584205, rs2678204, rs2725371, rs28375268, rs3212038, rs34483452, rs3764002, rs3935190, rs4290163, rs429358, rs4718964, rs4851283, rs61903695, rs6493498, rs6669341, rs6938973, rs7259070, rs7498665, rs76286777, rs862320, rs9843653, rs9902846
	WM			− 0.04	− 0.08 to 0.003	0.07			
	MR Egger			0.01	− 0.13 to 0.15	0.92			
	MR-PRESSO			** − 0.08**	** − 0.11 to − 0.04**	** < 0.001**			
aWHR	IVW	292	86	** − 0.06**	** − 0.09 to − 0.02**	**0.003**	951.3 (69.4%)	− 0.001 (0.20)	rs10445337, rs10891490, rs12415793, rs13107325, rs34312154, rs34322, rs3764002, rs41562, rs4420638, rs8054299
	WM			** − 0.04**	** − 0.08 to − 0.003**	**0.03**			
	MR Egger			− 0.005	− 0.09 to − 0.08	0.91			
	MR-PRESSO			** − 0.05**	** − 0.08 to − 0.02**	**0.001**			
VAT	IVW	5	38	− 0.12	− 0.26 to 0.02	0.10	21.1 (81.0%)	0.001 (0.99)	rs62048402
	WM			** − 0.16**	** − 0.27 to − 0.05**	**0.005**			
	MR Egger			− 0.13	− 1.47 to 1.21	0.85			
	MR-PRESSO			− 0.17	− 0.29 to − 0.04	0.08			
BMI^a^	IVW	471	72	** − 0.11**	** − 0.15 to − 0.07**	** < 0.001**	2377.4 (80.2%)	− 0.001 (0.15)	rs10182181, rs10510419, rs10761785, rs11713193, rs11781699, rs11782074, rs12150665, rs12981256, rs13049280, rs13107325, rs13186194, rs13263601, rs1421334, rs1501673, rs17182027, rs17367750, rs1884389, rs2273175, rs2289379, rs273512, rs274628, rs2984618, rs3101336, rs3754963, rs427943, rs4303732, rs4700608, rs6010784, rs6757852, rs7124681, rs738140, rs768023, rs805412, rs889398, rs9320823, rs9937053, rs9944219
	WM			** − 0.07**	** − 0.11 to − 0.03**	** < 0.001**			
	MR Egger			− 0.04	− 0.14 to 0.07	0.52			
	MR-PRESSO			** − 0.10**	** − 0.13 to − 0.07**	** < 0.001**			
WC^a^	IVW	325	57	** − 0.06**	** − 0.11 to − 0.008**	**0.02**	1706.9 (81.0%)	− 0.002 (0.11)	rs11787216, rs1191600, rs12103006, rs2253310, rs2306593, rs2470946, rs2568958, rs2584205, rs2678204, rs2725371, rs28375268, rs28489620, rs34483452, rs3764002, rs3935190, rs4290163, rs429358, rs4718964, rs4851283, rs61903695, rs6669341, rs6938973, rs7259070, rs7377083, rs76286777, rs862320, rs9843653, rs9902846
	WM			− 0.01	− 0.06 to 0.03	0.58			
	MR Egger			0.05	− 0.10 to 0.21	0.48			
	MR-PRESSO			** − 0.07**	** − 0.11 to − 0.02**	**0.002**			

Bold: *P* < 0.05

*aWHR* BMI-adjusted waist-to-hip ratio, *BMI* body mass index, *IVW* inverse-variance weighted, *MR-PRESSO* Mendelian randomisation Pleiotropy RESidual Sum and Outlier, *SNP* single nucleotide polymorphism, *VAI* visceral adiposity index, *WC* waist circumference, *WHR* waist-to-hip ratio, *WM* weighted median method

^a^Removing potentially pleiotropic SNPs (overlapped SNPs between BMI and WC), including rs10185199, rs11636611, rs11757278, rs11773362, rs12462975, rs1296328, rs13047416, rs1327259, rs1346841, rs1441264, rs1799923, rs215669, rs2439823, rs28350, rs3806114, rs3814883, rs40067, rs4017425, rs4148155, rs429343, rs4482463, rs6493498, rs6567160, rs7171864, rs7206608, rs7498665, rs756717, rs784257, rs8097672, rs879620, rs9294260, rs9478496, rs9926784

## Discussion

To our knowledge, this is the first study that provides evidence for the associations between obesity and memory using both cross-sectional and longitudinal analyses, as well as MR analyses. We have first analysed the associations of the greatest number (20) of obesity indicators in middle-aged and older participants with memory-related outcomes and generally shown associations of higher levels of obesity indicators with poorer baseline and follow-up memory function, as well as greater memory decline. Amongst these 20 indicators, five central obesity measures, specifically WHR, WHtR, ABSI, BRI and WHHR, showed a consistent association with both poor memory function and greater memory decline. Notably, WHHR was identified as the indicator with the strongest association with memory impairment in both cross-sectional and longitudinal analyses. Moreover, these associations were found to be weaker in those with higher education, underscoring education as an important effect modifier. Our finding suggests that interventions aimed at improving educational access and quality may play a role in mitigating the negative impact of obesity on cognitive health. Results of the MR analyses support a causal link between higher obesity indicator values and poorer cognitive performance. These results reinforce the importance of targeted prevention and intervention strategies for middle-aged and older individuals with obesity, especially central obesity, to delay or alleviate the progression of memory decline. The observed interaction between obesity, cognitive function and educational attainment highlights the need for a holistic approach aiming at both obesity and education in public health policy and individualised care strategies.

The results of our study are generally consistent with previous reports on the negative associations between markers of obesity, such as BMI and WC, and memory function [40–43]. The negative association could be explained by obesity-derived structural changes in the brain, including alterations in grey and white matter volumes, which might adversely affect the integrity of neural circuits involved in memory. Specifically, adipose tissue is an active endocrine organ that secretes hormones and adipokines. Dysregulation in the balance of these substances, such as leptin resistance, may affect brain structures, resulting in lower memory function [44]. Adipose tissue is also associated with increased production of pro-inflammatory cytokines. This systemic inflammation may extend to the brain and contribute to neuroinflammation, affecting brain structures associated with memory [45]. However, there were also some studies supporting the obesity paradox, suggesting that obesity might act as a protective factor for memory performance [12, 46, 47]. Our study found that those with general obesity assessed by weight and BSA had better memory function at baseline but showed no associations with follow-up memory function. Moreover, we also found that those with higher HC or PLM had better memory function at baseline, which might be explained by the beneficial effects of gluteofemoral fat [48] or by the role of muscle structure on brain structure and function [49]. However, these associations disappeared in our longitudinal analyses.

Previous studies on obesity and cognitive decline were inconsistent, with some showing an association between obesity and greater cognitive decline [50–53], whilst others found an association between obesity and slower cognitive decline [54–59] or no association [60–63]. Notably, cognition is multifaceted, encompassing not only memory but also other cognitive processes, including problem-solving, decision-making and executive functions. Results not distinguishing these cognitive facets would be challenging to interpret, and results related to memory might be obscured by the broader spectrum of cognition. Amongst the studies mentioned above, only three studies with small sample size (all with *n* ≤ 2134) explored the associations between obesity and memory decline, showing mixed results [51, 53, 54]. Our study of a well-established population-based Chinese cohort with large sample size (*N* = 16,370) can disentangle the specific influence of different obesity indicators on memory decline and provide new evidence that obesity was significantly associated with faster memory decline in middle-aged and older people. Additionally, our updated two-sample MR study also support the adverse effects of obesity on cognition.

Moreover, our results agreed that, amongst the 20 obesity indicators, higher values of WHR, WHtR, ABSI, BRI and WHHR were associated with lower memory function and faster memory decline consistently. Notably, all these indicators are metrics specifically targeting central obesity, highlighting the importance of considering central obesity rather than general obesity as a potential modifiable risk factor for memory decline. The findings might be due to the greater deleterious effects of visceral fat than subcutaneous fat [64]. Additionally, amongst these five indicators, WHHR was most relevant to memory, and the complexity involved in the calculation of ABSI and BRI should also be acknowledged. The strong associations observed with WHHR suggest that this simple indicator may serve as an important predictor for memory.

Only a few studies have reported the modifying role of education in the associations of obesity with memory. A Chinese study [12] and a western study [9] found no significant interaction, whilst two western studies showed that in participants with higher education, the association of obesity with cognitive dysfunction was weaker [11] or attenuated to null [10]. The protective effect of education might be attributed to its promotion of positive neuroplasticity [65]. Our study confirms and extends the evidence to an understudied population, supporting education could be an effect modifier in mitigating the adverse impact of obesity on memory function.

Our study had several limitations. Firstly, in GBCS, memory was assessed by a delayed 10-word recall test rather than a battery of cognitive tests, which was not feasible in large population-based study. However, the improved 10-word recall test has been shown to be straightforward and time-saving and has been validated as a sensitive and efficient tool for dementia screening, particularly assessing memory function, in developing countries [66] and in our previous papers [25, 26]. Secondly, we only focused on obesity indicators that could be obtained by simple anthropometric examinations or calculated by formula transformation. Although CT and MRI can provide more accurate estimate of the amount of fat stored in different adipose tissue compartments, their use is limited to clinical or laboratory settings but not for routine obesity diagnosis [6]. Thirdly, as all our participants were Chinese aged 50 years or above at baseline, generalisation of our results to younger populations and other ethnic groups might be limited. Fourthly, due to the lack of GWAS for obesity and memory in Chinese population, the genetic correlations between these 20 obesity indicators and memory function could not be examined.

## Conclusions

Our examination of 20 obesity indicators with memory-related outcomes in middle-aged and older participants generally showed associations of higher levels of obesity indicators with poorer memory function and greater memory decline, with the central obesity indicator WHHR identified as the most relevant indicator to memory. These associations were weaker in those with higher education levels, suggesting that socioeconomic factors may influence the impact of obesity on memory. Our findings emphasise the importance of managing obesity, especially central obesity, and improving educational access and quality to potentially mitigate the risk of obesity-related memory impairment in later life.

## Supplementary Information

Below is the link to the electronic supplementary material.

Supplementary file1 (DOCX 739 KB)

## Data Availability

Data that support findings from conventional observational study are restricted to researchers who have permission from the Guangzhou Biobank Cohort Study and so are not publicly available.

## Abbreviations

ABSI: A body shape index
BAI: Body adiposity index
BMI: Body mass index
BF%: Body fat percentage
BRI: Body roundness index
BSA: Body surface area
CI: Confidence interval
CMI: Cardiometabolic index
COGENT: Cognitive Genomics Consortium
CUN-BAE: Clínica Universidad de Navarra-Body Adiposity Estimator
CVAI: Chinese visceral adiposity index
DWRT: Delayed 10-word recall test
GBCS: Guangzhou Biobank Cohort Study
GEE: Generalised estimating equation
GHHARE: Guangzhou Health and Happiness Association for the Respectable Elders
GIANT: Genetic Investigation of Anthropometric Traits
GWAS: Genome-wide association studies
HC: Hip circumference
HR: Hazard ratio
IPAQ: International Physical Activity Questionnaire
IVW: Inverse-variance weighted
LD: Linkage disequilibrium
LAP: Lipid accumulation product
MR: Mendelian randomisation
MR-PRESSO: MR Pleiotropy RESidual Sum and Outlier
PFM: Predicted fat mass
PLM: Predicted lean mass
PPF: Predicted percent fat
SNP: Single nucleotide polymorphism
VAI: Visceral adiposity index
VAT: Visceral adipose tissue
WC: Waist circumference
WHHR: Waist-to-hip-to-height ratio
WHR: Waist-to-hip ratio
WHtR: Waist-to-height ratio
WM: Weighted median estimator
aWHR: WHR adjusted for BMI
