# Supplementary material for: Systematic analysis of associations between obesity and memory decline
Source: GeroScience. 2025 Jun 9;48(1):1143–60. doi: 10.1007/s11357-025-01725-3 (PMC12972487; doi:10.1007/s11357-025-01725-3)
Supplement: Supplementary file 1 — Supplementary file1 (DOCX 739 KB) [file 11357_2025_1725_MOESM1_ESM.docx]

**Supplementary method**

*Exposures*

We searched PubMed in title and abstract using keywords of “obesity indicators”, “obesity indices”, “obesity score”, “obesity markers” or “obesity index” up to 23^rd^ January 2024 and identified 1,487 studies. Generally, several indicators have been used to define obesity in the literature, including traditional obesity indicators such as body mass index (BMI), waist circumference (WC) and waist-to-hip ratio (WHR), and novel obesity indicators such as a body shape index (ABSI), lipid accumulation product (LAP) and visceral adiposity index (VAI). Among these indicators, some are obtained by simple anthropometric examinations, some are measured by CT scanning, bioelectrical impedance analysis or dual-energy X-ray absorptiometry, and some are calculated by formula transformation. Specifically, weight, WC, hip circumference (HC), abdominal circumference, neck circumference and calf circumference are the first category, fat mass and body fat percentage are the second category, and BMI, WHR, ABSI, LAP and VAI are the third category. In our study, we would focus on obesity indicators that are simple and easy to generalise broadly, i.e., category I and category III obesity indicators. Guangzhou Biobank Cohort Study (GBCS) included a face-to-face interview by trained nurses using a computer-assisted standardised questionnaire that included demographic characteristics, and assessment of anthropometric parameters and lipids. And anthropometric measures were measured with light indoor clothing and no shoes according to a standard protocol. After careful review, the following 20 indicators could be used in our study: weight, BMI, WC, HC, WHR, waist-to-height ratio (WHtR), LAP (1), ABSI (2), VAI (3), Chinese VAI (CVAI) (4), body roundness index (BRI) (5), conicity index (6), body adiposity index (BAI) (7), cardiometabolic index (CMI) (8), body surface area (BSA) (9), waist-to-hip-to-height ratio (WHHR), predicted fat mass (PFM) (10), predicted lean mass (PLM) (10), predicated percent fat (PPF) (10) and Clínica Universidad de Navarra-Body Adiposity Estimator (CUN-BAE) (11). Except for weight, WC and HC, which could be obtained directly from anthropometric examinations, other indicators would be calculated as follows:

$$BMI=\frac{weight (kg)}{{height (m)}^{2}}$$

$$WHR=\frac{WC (cm)}{HC (cm)}$$

$$WHtR=\frac{WC (cm)}{height (cm)}$$

$${LAP}_{men}=\left[ WC \left( cm \right)-65 \right]\times TG \left( {mmol}/l \right)$$

$${LAP}_{women}=[WC (cm)-58]\times TG (mmol/l)$$

$$ABSI=\frac{WC (m)}{{BMI}^{\frac{2}{3}}\times{height \left( m \right)}^{\frac{1}{2}}}$$

$${VAI}_{men}=(\frac{WC \left( cm \right)}{39.68+1.88\times BMI})\times(\frac{TG \left（ {mmol}/l \right）}{1.03})\times(\frac{1.31}{HDL \left（ {mmol}/l \right）})$$

$${VAI}_{women}=(\frac{WC \left( cm \right)}{36.58+1.89\times BMI})\times(\frac{TG \left（ {mmol}/l \right）}{0.81})\times(\frac{1.52}{HDL \left（ {mmol}/l \right）})$$

$${CVAI}_{men}=-267.93+0.69\times age\left( y \right)+0.03\times BMI+4.00\times WC \left( cm \right)+22.00\times Lg TG \left（ {mmol}/l \right）-16.32\times HDL \left（ {mmol}/l \right）$$

$${CVAI}_{women}=-187.32+1.71\times age\left( y \right)+4.32\times BMI+1.12\times WC \left( cm \right)+39.76\times Lg TG \left（ {mmol}/l \right）-11.66\times HDL \left（ {mmol}/l \right）$$

$$BRI=364.2-365.5\times\left\{ 1-\frac{{[WC {(m)}/{2\pi}]}^{2}}{{[0.5\times height \left( m \right)]}^{2}} \right\}^{\frac{1}{2}}$$

$$conicity index=\frac{WC (m)}{0.109\times\surd\frac{weight (kg)}{height (m)}}$$

$$BAI=\frac{HC (cm)}{{height (m)}^{1.5}}-18$$

$$CMI=\frac{TG \left（ {mmol}/l \right）}{HDL \left（ {mmol}/l \right）}\times WHtR$$

$$BSA=0.007184\times{height (cm)}^{0.725}\times{weight (kg)}^{0.425}$$

$$WHHR=\frac{WHR}{height (cm)}$$

$${PFM}_{men}=-18.592-0.009\times age \left( y \right)-0.080\times height \left( cm \right)+0.226\times weight \left( kg \right)+0.387\times WC \left( cm \right)+0.080\times Mexican-0.188\times Hispanic-0.483\times Black+1.050\times other ethnicity$$

$${PFM}_{women}=11.817+0.041\times age \left( y \right)-0.199\times height \left( cm \right)+0.610\times weight \left( kg \right)+0.044\times WC \left( cm \right)+0.388\times Mexican+0.073\times Hispanic-1.187\times Black+0.325\times other ethnicity$$

$${PLM}_{men}=19.363+0.001\times age \left( y \right)+0.064\times height \left( cm \right)+0.756\times weight \left( kg \right)-0.366\times WC \left( cm \right)-0.066\times Mexican+0.231\times Hispanic+0.432\times Black-1.007\times other ethnicity$$

$${PLM}_{women}=-10.683-0.039\times age \left( y \right)+0.186\times height \left( cm \right)+0.383\times weight \left( kg \right)-0.043\times WC \left( cm \right)-0.359\times Mexican-0.059\times Hispanic+1.085\times Black-0.34\times other ethnicity$$

$${PPF}_{men}=0.02+0.00\times age \left( y \right)-0.07\times height \left( cm \right)-0.08\times weight \left( kg \right)+0.48\times WC \left( cm \right)+0.32\times Mexican+0.02\times Hispanic-0.65\times Black+1.12\times other ethnicity$$

$${PPF}_{women}=50.46+0.07\times age \left( y \right)-0.26\times height \left( cm \right)+0.27\times weight \left( kg \right)+0.10\times WC \left( cm \right)+0.89\times Mexican+0.49\times Hispanic-1.57\times Black+0.43\times other ethnicity$$

$$CUN-BAE=-44.988+\left[ 0.503\times age \left( y \right) \right]+\left( 10.689\times sex \right)+\left( 3.172\times BMI \right)-\left( 0.026\times{BMI}^{2} \right)+\left( 0.181\times BMI\times sex \right)-\left[ 0.02\times BMI\times age \left( y \right) \right]-\left( 0.005\times{BMI}^{2}\times sex \right)+[0.00021\times{BMI}^{2}\times age \left( y \right)]$$

Note: HDL: high-density lipoprotein; TG: triglyceride; men=0 and women=1 for sex

Moreover, as the body build varies by ethnicity, we used the Chinese-specific cut-offs recommended by the World Health Organization (WHO) to define general obesity. Underweight was defined as BMI lower than 18.5 kg/m^2^, normal weight as BMI 18.5-24.9 kg/m^2^, overweight as BMI 25.0-27.4 kg/m^2^, and obesity as BMI ≥27.5 kg/m^2^ (12). Central obesity was defined as WC ≥90 cm in men and ≥80 cm in women (13). Higher WHR and WHtR were defined by established cut-offs, i.e., men ≥ 0.9 and women ≥ 0.8 for WHR (14) and ≥ 0.5 for WHtR (15).

**References**

1. Kahn HS. The "lipid accumulation product" performs better than the body mass index for recognizing cardiovascular risk: a population-based comparison. BMC Cardiovasc Disord. 2005;5:26.

2. Krakauer NY, Krakauer JC. A new body shape index predicts mortality hazard independently of body mass index. PLoS One. 2012;7(7):e39504.

3. Amato MC, Giordano C, Galia M, Criscimanna A, Vitabile S, Midiri M, et al. Visceral Adiposity Index: a reliable indicator of visceral fat function associated with cardiometabolic risk. Diabetes Care. 2010;33(4):920-2.

4. Xia MF, Chen Y, Lin HD, Ma H, Li XM, Aleteng Q, et al. A indicator of visceral adipose dysfunction to evaluate metabolic health in adult Chinese. Sci Rep. 2016;6:38214.

5. Thomas DM, Bredlau C, Bosy-Westphal A, Mueller M, Shen W, Gallagher D, et al. Relationships between body roundness with body fat and visceral adipose tissue emerging from a new geometrical model. Obesity (Silver Spring). 2013;21(11):2264-71.

6. Valdez R, Seidell JC, Ahn YI, Weiss KM. A new index of abdominal adiposity as an indicator of risk for cardiovascular disease. A cross-population study. Int J Obes Relat Metab Disord. 1993;17(2):77-82.

7. Bergman RN, Stefanovski D, Buchanan TA, Sumner AE, Reynolds JC, Sebring NG, et al. A better index of body adiposity. Obesity (Silver Spring). 2011;19(5):1083-9.

8. Wakabayashi I, Daimon T. The "cardiometabolic index" as a new marker determined by adiposity and blood lipids for discrimination of diabetes mellitus. Clin Chim Acta. 2015;438:274-8.

9. Du Bois D, Du Bois EF. A formula to estimate the approximate surface area if height and weight be known. 1916. Nutrition. 1989;5(5):303-11; discussion 12-3.

10. Lee DH, Keum N, Hu FB, Orav EJ, Rimm EB, Sun Q, et al. Development and validation of anthropometric prediction equations for lean body mass, fat mass and percent fat in adults using the National Health and Nutrition Examination Survey (NHANES) 1999-2006. Br J Nutr. 2017;118(10):858-66.

11. Gomez-Ambrosi J, Silva C, Galofre JC, Escalada J, Santos S, Millan D, et al. Body mass index classification misses subjects with increased cardiometabolic risk factors related to elevated adiposity. Int J Obes (Lond). 2012;36(2):286-94.

12. Consultation WHOE. Appropriate body-mass index for Asian populations and its implications for policy and intervention strategies. Lancet (London, England). 2004;363(9403):157-63.

13. Alberti KG, Eckel RH, Grundy SM, Zimmet PZ, Cleeman JI, Donato KA, et al. Harmonizing the metabolic syndrome: a joint interim statement of the International Diabetes Federation Task Force on Epidemiology and Prevention; National Heart, Lung, and Blood Institute; American Heart Association; World Heart Federation; International Atherosclerosis Society; and International Association for the Study of Obesity. Circulation. 2009;120(16):1640-5.

14. Huxley R, James WP, Barzi F, Patel JV, Lear SA, Suriyawongpaisal P, et al. Ethnic comparisons of the cross-sectional relationships between measures of body size with diabetes and hypertension. Obes Rev. 2008;9 Suppl 1:53-61.

15. Browning LM, Hsieh SD, Ashwell M. A systematic review of waist-to-height ratio as a screening tool for the prediction of cardiovascular disease and diabetes: 0.5 could be a suitable global boundary value. Nutr Res Rev. 2010;23(2):247-69.

Table S1. Study details for the GWAS of exposures and outcome.

| **Phenotype** | **Definition** | **Unit** | **Year** | **Consortium** | **Sample size** | **Mean age** | **Adjust** | **Pubmed ID** | **GWAS ID** |
| --- | --- | --- | --- | --- | --- | --- | --- | --- | --- |
| **Exposures** | | | | | | | | | |
| BMI | weight (kg) / height (m) squared | kg/m^2^ | 2019 | GIANT consortium, UK Biobank | 806,834 | NA | NA | 30239722 | NA |
| WC | waist circumference | cm | 2018 | UK Biobank | 462,166 | NA | NA | NA | ukb-b-9405 |
| aWHR | waist-to-hip ratio adjusted for body mass index | - | 2019 | GIANT consortium, UK Biobank | 697,734 | NA | NA | 30239722 | NA |
| VAT | visceral adipose tissue volumes estimated using neural-network based methods from the Dixon segmentation | L | 2021 | UK Biobank | 32,860 | 63.9 (7.52) years | genetic sex, age, age^2^, the first 10 PCs of genetic ancestry, scaled scan date, scaled scan time, study centre, and genetic relatedness | 34128465 | ebi-a-GCST90016671 |
| **Outcome** | | | | | | | | | |
| Cognitive performance | The score on a test of verbal cognition or Henmon-Nelson test of mental ability | - | 2018 | COGENT consortium, UK Biobank | 257,841 | At least 30 years | unadjusted | 30038396 | ebi-a-GCST006572 |

aWHR: adjusted waist-to-hip ratio; BMI: body mass index; COGENT, Cognitive Genomics Consortium; GIANT, Genetic Investigation of Anthropometric Traits; GWAS, genome-wide association study; VAT: visceral adiposity tissue; WC: waist circumference; PC, principal component

Table S2. Associations of baseline obesity indicators with memory function at baseline.

|  | *N* | Adjusted mean differences β (95% CI) in baseline DWRT score | | |  |
| --- | --- | --- | --- | --- | --- |
|  |  | Model 1^a^ | Model 2^b^ | Model 3^c^ | P for non-linearity^d^ |
| Weight, kg | 27,979 | 0.006 (0.004 to 0.008)^***^ | 0.004 (0.002 to 0.007)^***^ | 0.003 (0.001 to 0.006)^**^ | 0.48 |
| BMI, kg/m^2^ | 27,979 | 0.0005 (-0.006 to 0.007) | -0.003 (-0.009 to 0.003) | 0.001 (-0.005 to 0.007) | 0.27 |
| WC, cm | 27,979 | -0.01 (-0.02 to -0.01)^***^ | -0.006 (-0.008 to -0.003)^***^ | -0.002 (-0.005 to 0) | 0.86 |
| HC, cm | 27,979 | 0.006 (0.002 to 0.009)^**^ | 0.006 (0.003 to 0.009)^***^ | 0.007 (0.003 to 0.01)^***^ | 0.20 |
| WHR | 27,979 | -3.30 (-3.62 to -2.99)^***^ | -1.82 (-2.15 to -1.49)^***^ | -1.12 (-1.44 to -0.80)^***^ | 0.83 |
| WHtR | 27,979 | -3.03 (-3.41 to -2.66)^***^ | -1.54 (-1.91 to -1.16)^***^ | -0.69 (-1.06 to -0.32)^***^ | 0.69 |
| LAP | 27,650 | -0.002 (-0.002 to -0.001)^***^ | -0.001 (-0.002 to -0.001)^***^ | -0.0005 (-0.001 to 0.0001) | 0.50 |
| ABSI | 27,979 | -56.48 (-60.82 to -52.14)^***^ | -25.74 (-30.34 to -21.13)^***^ | -14.65 (-19.17 to -10.12)^***^ | 0.05 |
| VAI | 27,979 | -0.006 (-0.02 to 0.01) | -0.02 (-0.03 to -0.004)^**^ | -0.007 (-0.02 to 0.004) | 0.20 |
| CVAI | 27,979 | -0.003 (-0.004 to -0.003)^***^ | -0.001 (-0.001 to -0.0002)^**^ | -0.0002 (-0.0007 to 0.0004) | 0.49 |
| BRI | 27,979 | -0.16 (-0.18 to -0.14)^***^ | -0.08 (-0.10 to -0.06)^***^ | -0.04 (-0.06 to -0.02)^***^ | 0.92 |
| Conicity index | 27,979 | -18.07 (-19.58 to -16.56)^***^ | -9.68 (-11.29 to -8.07)^***^ | -5.83 (-7.41 to -4.25)^***^ | 0.03 |
| BAI | 27,979 | -0.01 (-0.02 to -0.007)^***^ | -0.01 (-0.02 to -0.004)^**^ | 0.0004 (-0.005 to 0.006) | 0.22 |
| CMI | 27,979 | -0.04 (-0.08 to -0.005)^*^ | -0.04 (-0.08 to -0.01)^*^ | -0.02 (-0.05 to 0.02) | 0.45 |
| BSA | 27,979 | 0.54 (0.39 to 0.69)^***^ | 0.52 (0.37 to 0.69)^***^ | 0.34 (0.17 to 0.50)^***^ | 0.67 |
| WHHR | 27,979 | -555.72 (-601.09 to -510.34)^***^ | -333.10 (-379.51 to -286.69)^***^ | -197.87 (-243.45 to -152.30)^***^ | <0.001 |
| PFM | 27,979 | 0.009 (0.006 to 0.01)^***^ | 0.0006 (-0.003 to 0.005) | 0.001 (-0.002 to 0.005) | 0.13 |
| PLM | 27,979 | 0.003 (0.0004 to 0.006)^*^ | 0.02 (0.01 to 0.02)^***^ | 0.01 (0.008 to 0.02)^***^ | 0.09 |
| PPF | 27,979 | 0.005 (0.002 to 0.008)^**^ | -0.01 (-0.02 to -0.007)^***^ | -0.006 (-0.01 to 0.0006) | 0.47 |
| CUN-BAE | 27,979 | 0.002 (-0.001 to 0.005) | -0.002 (-0.007 to 0.003) | 0.001 (-0.004 to 0.006) | 0.03 |
| Weight z-score | 27,979 | 0.06 (0.04 to 0.08)^***^ | 0.04 (0.02 to 0.06)^***^ | 0.03 (0.01 to 0.05)^**^ |  |
| BMI z-score | 27,979 | 0.002 (-0.02 to 0.02) | -0.01 (-0.03 to 0.01) | 0.004 (-0.02 to 0.02) |  |
| WC z-score | 27,979 | -0.13 (-0.15 to -0.11)^***^ | -0.05 (-0.07 to -0.03)^***^ | -0.02 (-0.04 to 0.00005) |  |
| HC z-score | 27,979 | 0.04 (0.01 to 0.06)^**^ | 0.04 (0.02 to 0.06)^***^ | 0.04 (0.02 to 0.06)^***^ |  |
| WHR z-score | 27,979 | -0.22 (-0.25 to -0.20)^***^ | -0.12 (-0.15 to -0.10)^***^ | -0.08 (-0.10 to -0.05)^***^ |  |
| WHtR z-score | 27,979 | -0.17 (-0.19 to -0.15)^***^ | -0.09 (-0.11 to -0.07)^***^ | -0.04 (-0.06 to -0.02)^***^ |  |
| LAP z-score | 27,650 | -0.06 (-0.08 to -0.04)^***^ | -0.04 (-0.06 to -0.02)^***^ | -0.02 (-0.04 to 0.005) |  |
| ABSI z-score | 27,979 | -0.28 (-0.30 to -0.26)^***^ | -0.13 (-0.15 to -0.10)^***^ | -0.07 (-0.09 to -0.05)^***^ |  |
| VAI z-score | 27,979 | -0.01 (-0.03 to 0.01) | -0.03 (-0.05 to -0.01)^**^ | -0.01 (-0.03 to 0.01) |  |
| CVAI z-score | 27,979 | -0.14 (-0.16 to -0.12)^***^ | -0.03 (-0.05 to -0.01)^**^ | -0.006 (-0.03 to 0.02) |  |
| BRI z-score | 27,979 | -0.18 (-0.20 to -0.15)^***^ | -0.09 (-0.11 to -0.07)^***^ | -0.04 (-0.06 to -0.02)^***^ |  |
| Conicity index z-score | 27,979 | -0.26 (-0.28 to -0.24)^***^ | -0.14 (-0.16 to -0.11)^***^ | -0.08 (-0.11 to -0.06)^***^ |  |
| BAI z-score | 27,979 | -0.05 (-0.07 to -0.03)^***^ | -0.04 (-0.07 to -0.02)^**^ | 0.002 (-0.02 to 0.03) |  |
| CMI z-score | 27,979 | -0.02 (-0.05 to -0.002)^*^ | -0.03 (-0.05 to -0.006)^*^ | -0.01 (-0.03 to 0.01) |  |
| BSA z-score | 27,979 | 0.08 (0.06 to 0.10)^***^ | 0.08 (0.05 to 0.10)^***^ | 0.05 (0.03 to 0.07)^***^ |  |
| WHHR z-score | 27,979 | -0.26 (-0.28 to -0.24)^***^ | -0.16 (-0.18 to -0.14)^***^ | -0.09 (-0.11 to -0.07)^***^ |  |
| PFM z-score | 27,979 | 0.06 (0.04 to 0.08)^***^ | 0.004 (-0.02 to 0.03) | 0.01 (-0.01 to 0.03) |  |
| PLM z-score | 27,979 | 0.02 (0.003 to 0.05)^*^ | 0.14 (0.11 to 0.18)^***^ | 0.10 (0.06 to 0.13)^***^ |  |
| PPF z-score | 27,979 | 0.03 (0.01 to 0.05)^**^ | -0.10 (-0.15 to -0.05)^***^ | -0.04 (-0.09 to 0.004) |  |
| CUN-BAE z-score | 27,979 | 0.01 (-0.01 to 0.03) | -0.02 (-0.05 to 0.02) | 0.007 (-0.03 to 0.04) |  |
| BMI groups |  |  |  |  |  |
| Underweight | 1,229 | -0.21 (-0.31 to -0.11)^***^ | -0.11 (-0.21 to -0.009)^*^ | -0.06 (-0.16 to 0.04) |  |
| Normal | 17,247 | Ref. (0) | Ref. (0) | Ref. (0) |  |
| Overweight | 5,915 | -0.03 (-0.08 to 0.02) | -0.03 (-0.08 to 0.03) | 0.002 (-0.05 to 0.05) |  |
| Obese | 3,588 | -0.06 (-0.12 to 0.01) | -0.06 (-0.12 to 0.01) | 0.005 (-0.06 to 0.07) |  |
| WC groups |  |  |  |  |  |
| Normal | 18,341 | Ref. (0) | Ref. (0) | Ref. (0) |  |
| High | 9,638 | -0.19 (-0.23 to -0.14)^***^ | -0.11 (-0.16 to -0.07)^***^ | -0.03 (-0.08 to 0.01) |  |
| BMI & WC groups |  |  |  |  |  |
| Non-obese | 15,751 | Ref. (0) | Ref. (0) | Ref. (0) |  |
| High BMI | 2,590 | 0.13 (0.05 to 0.20)^***^ | 0.07 (-0.01 to 0.14) | 0.04 (-0.03 to 0.11) |  |
| High WC | 2,725 | -0.26 (-0.34 to -0.19)^***^ | -0.13 (-0.21 to -0.06)^***^ | -0.05 (-0.12 to 0.02) |  |
| High BMI & WC | 6,913 | -0.14 (-0.19 to -0.08)^***^ | -0.10 (-0.15 to -0.04)^***^ | -0.02 (-0.07 to 0.03) |  |
| WHR groups |  |  |  |  |  |
| Normal | 7,709 | Ref. (0) | Ref. (0) | Ref. (0) |  |
| High | 20,270 | -0.22 (-0.27 to -0.17)^***^ | -0.19 (-0.24 to -0.15)^***^ | -0.12 (-0.17 to -0.08)^***^ |  |
| WHtR groups |  |  |  |  |  |
| Normal | 13,440 | Ref. (0) | Ref. (0) | Ref. (0) |  |
| High | 14,539 | -0.31 (-0.35 to -0.27)^***^ | -0.16 (-0.20 to -0.11)^***^ | -0.07 (-0.11 to -0.03)^**^ |  |

ABSI: a body shape index; BAI: body adiposity index; BMI: body mass index; BRI: body roundness index; BSA: body surface area; CI: confidence interval; CMI: cardiometabolic index; CUN-BAE: Clínica Universidad de Navarra-Body Adiposity Estimator; CVAI: Chinese visceral adiposity index; DWRT: Delayed Word Recall Test; HC, hip circumference; LAP: lipid accumulation product; PFM: predicted fat mass; PLM: predicted lean mass; PPF: predicated percent fat; Ref, reference; VAI: visceral adiposity index; WC: waist circumference; WHHR: waist-to-hip-to-height ratio; WHR: waist-to-hip ratio; WHtR: waist-to-height ratio

^a^: Unadjusted

^b^: Adjusted for sex and age

^c^: Additionally adjusted for education, occupation, personal income, physical activity, drinking, smoking and self-rated health

^d^: In model 3

^*^P <0.05, ^**^P <0.01, ^***^P <0.001

Underweight: BMI <18.5 kg/m^2^; Normal: 18.5 kg/m^2^ ≤ BMI <25 kg/m^2^; Overweight: 25 kg/m^2^ ≤ BMI < 27.5 kg/m^2^; Obese: BMI ≥ 27.5 kg/m^2^; High BMI: BMI ≥ 25 kg/m^2^

Normal WC: <90 cm for men, <80 cm for women; High WC: ≥90 cm for men, ≥80 cm for women

Normal WHR: <0.9 for men, <0.8 for women; High WHR: ≥0.9 for men, ≥0.8 for women

Normal WHtR: <0.5; High WHR: ≥0.5

Table S3. Associations of baseline obesity indicators with memory function at baseline by education.

|  | *N* | Adjusted mean differences β (95% CI) in baseline DWRT score | | |
| --- | --- | --- | --- | --- |
|  |  | Model 1^a^ | Model 2^b^ | Model 3^c^ |
| **Weight z-score** |  |  |  |  |
| Primary or less | 11,990 | 0.10 (0.07 to 0.13)^***^ | 0.07 (0.03 to 0.10)^***^ | 0.05 (0.02 to 0.08)^**^ |
| Secondary or more | 15,989 | -0.04 (-0.06 to -0.009)^**^ | 0.03 (0.005 to 0.06)^*^ | 0.02 (-0.007 to 0.05) |
| P for interaction |  | <0.001 | 0.005 | 0.03 |
| **BMI z-score** |  |  |  |  |
| Primary or less | 11,990 | 0.06 (0.03 to 0.09)^***^ | 0.03 (-0.004 to 0.06) | 0.01 (-0.02 to 0.05) |
| Secondary or more | 15,989 | 0.01 (-0.02 to 0.04) | 0.002 (-0.03 to 0.03) | -0.003 (-0.03 to 0.02) |
| P for interaction |  | 0.03 | 0.28 | 0.42 |
| **WC z-score** |  |  |  |  |
| Primary or less | 11,990 | -0.04 (-0.07 to -0.004) | -0.01 (-0.04 to 0.02) | -0.01 (-0.04 to 0.02) |
| Secondary or more | 15,989 | -0.13 (-0.15 to -0.10)^***^ | -0.02 (-0.05 to 0.005) | -0.02 (-0.05 to 0.003) |
| P for interaction |  | <0.001 | 0.31 | 0.53 |
| **HC z-score** |  |  |  |  |
| Primary or less | 11,990 | 0.09 (0.06 to 0.12)^***^ | 0.07 (0.04 to 0.10)^***^ | 0.06 (0.03 to 0.09)^***^ |
| Secondary or more | 15,989 | 0.02 (-0.01 to 0.05) | 0.03 (0.005 to 0.06)^*^ | 0.03 (-0.001 to 0.05) |
| P for interaction |  | <0.001 | 0.10 | 0.10 |
| **WHR z-score** |  |  |  |  |
| Primary or less | 11,990 | -0.14 (-0.18 to -0.11)^***^ | -0.08 (-0.12 to -0.05)^***^ | -0.08 (-0.12 to -0.05)^***^ |
| Secondary or more | 15,989 | -0.20 (-0.22 to -0.17)^***^ | -0.07 (-0.10 to -0.04)^***^ | -0.07 (-0.10 to -0.04)^***^ |
| P for interaction |  | 0.02 | 0.89 | 0.49 |
| **WHtR z-score** |  |  |  |  |
| Primary or less | 11,990 | -0.07 (-0.10 to -0.04)^***^ | -0.04 (-0.07 to -0.004)^*^ | -0.04 (-0.07 to -0.004)^*^ |
| Secondary or more | 15,989 | -0.10 (-0.13 to -0.07)^***^ | -0.05 (-0.07 to -0.02)^**^ | -0.04 (-0.07 to -0.01)^**^ |
| P for interaction |  | 0.12 | 0.81 | 0.71 |
| **LAP z-score** |  |  |  |  |
| Primary or less | 11,859 | -0.004 (-0.04 to 0.03) | -0.01 (-0.04 to 0.02) | -0.01 (-0.04 to 0.02) |
| Secondary or more | 15,791 | -0.03 (-0.06 to -0.005)^*^ | -0.02 (-0.05 to 0.01) | -0.02 (-0.05 to 0.01) |
| P for interaction |  | 0.17 | 0.97 | 0.89 |
| **ABSI z-score** |  |  |  |  |
| Primary or less | 11,990 | -0.19 (-0.22 to -0.16)^***^ | -0.10 (-0.13 to -0.06)^***^ | -0.08 (-0.11 to -0.05)^***^ |
| Secondary or more | 15,989 | -0.22 (-0.25 to -0.19)^***^ | -0.08 (-0.11 to -0.05)^***^ | -0.06 (-0.09 to -0.03)^***^ |
| P for interaction |  | 0.21 | 0.27 | 0.26 |
| **VAI z-score** |  |  |  |  |
| Primary or less | 11,990 | 0.01 (-0.02 to 0.04) | -0.02 (-0.05 to 0.02) | -0.01 (-0.04 to 0.02) |
| Secondary or more | 15,989 | 0.01 (-0.02 to 0.04) | -0.01 (-0.04 to 0.02) | -0.01 (-0.04 to 0.02) |
| P for interaction |  | 0.91 | 0.61 | 0.67 |
| **CVAI z-score** |  |  |  |  |
| Primary or less | 11,990 | -0.05 (-0.08 to -0.02)^**^ | 0.01 (-0.02 to 0.05) | 0.006 (-0.03 to 0.04) |
| Secondary or more | 15,989 | -0.06 (-0.09 to -0.04)^***^ | -0.01 (-0.04 to 0.02) | -0.01 (-0.04 to 0.02) |
| P for interaction |  | 0.47 | 0.83 | 0.92 |
| **BRI z-score** |  |  |  |  |
| Primary or less | 11,990 | -0.07 (-0.10 to -0.04)^***^ | -0.04 (-0.07 to -0.006)^*^ | -0.04 (-0.07 to -0.005)^*^ |
| Secondary or more | 15,989 | -0.11 (-0.14 to -0.08)^***^ | -0.05 (-0.08 to -0.02)^**^ | -0.04 (-0.07 to -0.01)^**^ |
| P for interaction |  | 0.09 | 0.84 | 0.76 |
| **Conicity index z-score** |  |  |  |  |
| Primary or less | 11,990 | -0.20 (-0.23 to -0.17)^***^ | -0.13 (-0.16 to -0.09)^***^ | -0.10 (-0.14 to -0.07)^***^ |
| Secondary or more | 15,989 | -0.13 (-0.16 to -0.10)^***^ | -0.09 (-0.12 to -0.06)^***^ | -0.07 (-0.10 to -0.04)^***^ |
| P for interaction |  | 0.001 | 0.004 | 0.01 |
| **BAI z-score** |  |  |  |  |
| Primary or less | 11,990 | 0.01 (-0.02 to 0.04) | 0.008 (-0.03 to 0.04) | 0.01 (-0.02 to 0.05) |
| Secondary or more | 15,989 | 0.08 (0.05 to 0.11)^***^ | -0.01 (-0.05 to 0.01) | -0.008 (-0.04 to 0.02) |
| P for interaction |  | 0.002 | 0.26 | 0.51 |
| **CMI z-score** |  |  |  |  |
| Primary or less | 11,990 | 0.001 (-0.03 to 0.03) | -0.01 (-0.05 to 0.02) | -0.01 (-0.04 to 0.02) |
| Secondary or more | 15,989 | -0.02 (-0.05 to 0.005) | -0.008 (-0.03 to 0.02) | -0.007 (-0.03 to 0.02) |
| P for interaction |  | 0.28 | 0.89 | 0.85 |
| **BSA z-score** |  |  |  |  |
| Primary or less | 11,990 | 0.11 (0.08 to 0.14)^***^ | 0.10 (0.06 to 0.13)^***^ | 0.08 (0.04 to 0.11)^***^ |
| Secondary or more | 15,989 | -0.06 (-0.08 to -0.03)^***^ | 0.05 (0.02 to 0.08)^**^ | 0.04 (0.005 to 0.07)^*^ |
| P for interaction |  | <0.001 | <0.001 | 0.006 |
| **WHHR z-score** |  |  |  |  |
| Primary or less | 11,990 | -0.17 (-0.20 to -0.14)^***^ | -0.12 (-0.15 to -0.08)^***^ | -0.11 (-0.14 to -0.08)^***^ |
| Secondary or more | 15,989 | -0.15 (-0.18 to -0.13)^***^ | -0.09 (-0.12 to -0.06)^***^ | -0.08 (-0.11 to -0.05)^***^ |
| P for interaction |  | 0.40 | 0.07 | 0.049 |
| **PFM z-score** |  |  |  |  |
| Primary or less | 11,990 | 0.10 (0.07 to 0.13)^***^ | 0.05 (0.01 to 0.08)^*^ | 0.03 (-0.006 to 0.07) |
| Secondary or more | 15,989 | 0.13 (0.10 to 0.16)^***^ | 0.004 (-0.03 to 0.04) | -0.005 (-0.04 to 0.03) |
| P for interaction |  | 0.15 | 0.87 | 0.83 |
| **PLM z-score** |  |  |  |  |
| Primary or less | 11,990 | 0.05 (0.02 to 0.09)^**^ | 0.17 (0.11 to 0.23)^***^ | 0.13 (0.07 to 0.19)^***^ |
| Secondary or more | 15,989 | -0.13 (-0.16 to -0.11)^***^ | 0.11 (0.06 to 0.16)^***^ | 0.08 (0.04 to 0.13)^**^ |
| P for interaction |  | <0.001 | <0.001 | 0.003 |
| **PPF z-score** |  |  |  |  |
| Primary or less | 11,990 | 0.06 (0.03 to 0.10)^**^ | 0.009 (-0.06 to 0.08) | -0.01 (-0.08 to 0.06) |
| Secondary or more | 15,989 | 0.18 (0.15 to 0.21)^***^ | -0.06 (-0.12 to 0.001) | -0.06 (-0.13 to -0.004)^*^ |
| P for interaction |  | <0.001 | 0.01 | 0.13 |
| **CUN-BAE z-score** |  |  |  |  |
| Primary or less | 11,990 | 0.05 (0.02 to 0.09)^**^ | 0.05 (-0.005 to 0.10) | 0.03 (-0.03 to 0.08) |
| Secondary or more | 15,989 | 0.16 (0.13 to 0.18)^***^ | 0.004 (-0.04 to 0.05) | -0.006 (-0.05 to 0.04) |
| P for interaction |  | <0.001 | 0.03 | 0.19 |

ABSI: a body shape index; BMI: body mass index; BRI: body roundness index; CI: confidence interval; CVAI: Chinese visceral adiposity index; DWRT: Delayed Word Recall Test; HC, hip circumference; LAP: lipid accumulation product; Ref, reference; VAI: visceral adiposity index; WC: waist circumference; WHR: waist-to-hip ratio; WHtR: waist-to-height ratio

^a^: Unadjusted

^b^: Adjusted for sex and age

^c^: Additionally adjusted for occupation, personal income, physical activity, drinking, smoking and self-rated health

^*^P <0.05, ^**^P <0.01, ^***^P <0.001

Underweight: BMI <18.5 kg/m^2^; Normal: 18.5 kg/m^2^ ≤ BMI <25 kg/m^2^; Overweight: 25 kg/m^2^ ≤ BMI < 27.5 kg/m^2^; Obese: BMI ≥ 27.5 kg/m^2^; High BMI: BMI ≥ 25 kg/m^2^

Normal WC: <90 cm for men, <80 cm for women; High WC: ≥90 cm for men, ≥80 cm for women

Normal WHR: <0.9 for men, <0.8 for women; High WHR: ≥0.9 for men, ≥0.8 for women

Normal WHtR: <0.5; High WHR: ≥0.5

Table S4. Associations of baseline obesity indicators with memory function at follow-up.

|  | *N* | Adjusted mean differences β (95% CI) in follow-up DWRT score | | |  |
| --- | --- | --- | --- | --- | --- |
|  |  | Model 1^a^ | Model 2^b^ | Model 3^c^ | P for non-linearity^d^ |
| Weight, kg | 16,370 | -0.001 (-0.005 to 0.002) | -0.001 (-0.004 to 0.002) | -0.001 (-0.005 to 0.002) | 0.72 |
| BMI, kg/m^2^ | 16,370 | -0.02 (-0.03 to -0.01)^***^ | -0.01 (-0.02 to -0.001)^*^ | -0.01 (-0.02 to 0.0002) | 0.78 |
| WC, cm | 16,370 | -0.01 (-0.01 to -0.01)^***^ | -0.01 (-0.01 to -0.002)^**^ | -0.004 (-0.01 to -0.001)^**^ | 0.92 |
| HC, cm | 16,370 | -0.001 (-0.005 to 0.004) | 0.0004 (-0.004 to 0.005) | -0.001 (-0.005 to 0.004) | 0.81 |
| WHR | 16,370 | -1.94 (-2.41 to -1.47)^***^ | -1.19 (-1.65 to -0.72)^***^ | -0.87 (-1.31 to -0.42)^***^ | 0.57 |
| WHtR | 16,370 | -2.00 (-2.53 to -1.47)^***^ | -1.11 (-1.64 to -0.58)^***^ | -0.85 (-1.36 to -0.34)^**^ | 0.27 |
| LAP | 16,195 | -0.001 (-0.002 to -0.0004)^**^ | -0.0005 (-0.001 to 0.0004) | -0.0004 (-0.001 to 0.0005) | 0.61 |
| ABSI | 16,370 | -20.84 (-27.33 to -14.35)^***^ | -10.98 (-17.42 to -4.54)^**^ | -7.56 (-13.76 to -1.36)^*^ | 0.65 |
| VAI | 16,370 | -0.01 (-0.02 to 0.01) | 0.001 (-0.02 to 0.02) | 0.002 (-0.01 to 0.02) | 0.81 |
| CVAI | 16,370 | -0.001 (-0.002 to -0.0004)^**^ | -0.001 (-0.001 to 0.0002) | -0.0004 (-0.001 to 0.0003) | 0.04 |
| BRI | 16,370 | -0.11 (-0.13 to -0.08)^***^ | -0.06 (-0.09 to -0.03)^***^ | -0.05 (-0.07 to -0.02)^**^ | 0.30 |
| Conicity index | 16,370 | -5.75 (-8.03 to -3.46)^***^ | -2.80 (-5.07 to -0.53)^*^ | -1.55 (-3.74 to 0.63) | 0.76 |
| BAI | 16,370 | -0.02 (-0.03 to -0.009)^***^ | -0.007 (-0.02 to 0.001) | -0.006 (-0.01 to 0.002) | 0.70 |
| CMI | 16,370 | -0.02 (-0.07 to 0.03) | 0.006 (-0.04 to 0.05) | 0.01 (-0.04 to 0.06) | 0.49 |
| BSA | 16,370 | 0.13 (-0.11 to 0.36) | 0.04 (-0.19 to 0.27) | -0.03 (-0.25 to 0.20) | 0.80 |
| WHHR | 16,370 | -327.48 (-393.55 to -261.41)^***^ | -194.71 (-260.32 to -129.11)^***^ | -138.12 (-201.33 to -74.90)^***^ | 0.02 |
| PFM | 16,370 | -0.007 (-0.01 to -0.002)^**^ | -0.005 (-0.01 to 0.0005) | -0.004 (-0.01 to -0.001) | 0.19 |
| PLM | 16,370 | 0.006 (-0.001 to 0.01) | 0.003 (-0.004 to 0.01) | 0.0003 (-0.007 to 0.007) | 0.07 |
| PPF | 16,370 | -0.02 (-0.03 to -0.01)^***^ | -0.01 (-0.02 to -0.004)^**^ | -0.01 (-0.02 to -0.001)^*^ | 0.04 |
| CUN-BAE | 16,370 | -0.01 (-0.02 to -0.005)^**^ | -0.007 (-0.01 to -0.0003)^*^ | -0.006 (-0.01 to 0.0005) | 0.08 |
| Weight z-score | 16,370 | -0.01 (-0.04 to 0.02) | -0.01 (-0.04 to 0.02) | -0.01 (-0.04 to 0.02) |  |
| BMI z-score | 16,370 | -0.06 (-0.08 to -0.03)^***^ | -0.03 (-0.06 to -0.003)^*^ | -0.03 (-0.06 to 0.001) |  |
| WC z-score | 16,370 | -0.09 (-0.12 to -0.06)^***^ | -0.05 (-0.08 to -0.02)^**^ | -0.04 (-0.07 to -0.01)^**^ |  |
| HC z-score | 16,370 | -0.005 (-0.03 to 0.02) | 0.002 (-0.03 to 0.03) | -0.005 (-0.03 to 0.02) |  |
| WHR z-score | 16,370 | -0.13 (-0.16 to -0.10)^***^ | -0.08 (-0.11 to -0.05)^***^ | -0.06 (-0.09 to -0.03)^***^ |  |
| WHtR z-score | 16,370 | -0.11 (-0.14 to -0.08)^***^ | -0.06 (-0.09 to -0.03)^***^ | -0.05 (-0.08 to -0.02)^**^ |  |
| LAP z-score | 16,195 | -0.04 (-0.08 to -0.01)^**^ | -0.02 (-0.05 to 0.01) | -0.01 (-0.04 to 0.02) |  |
| ABSI z-score | 16,370 | -0.10 (-0.13 to -0.07)^***^ | -0.05 (-0.09 to -0.02)^**^ | -0.04 (-0.07 to -0.01)^*^ |  |
| VAI z-score | 16,370 | -0.01 (-0.04 to 0.02) | 0.001 (-0.03 to 0.03) | 0.003 (-0.03 to 0.03) |  |
| CVAI z-score | 16,370 | -0.05 (-0.08 to -0.02)^**^ | -0.02 (-0.05 to 0.01) | -0.02 (-0.05 to 0.01) |  |
| BRI z-score | 16,370 | -0.12 (-0.15 to -0.09)^***^ | -0.07 (-0.10 to -0.04)^***^ | -0.05 (-0.08 to -0.02)^**^ |  |
| Conicity index z-score | 16,370 | -0.08 (-0.11 to -0.05)^***^ | -0.04 (-0.07 to -0.01)^*^ | -0.02 (-0.05 to 0.01) |  |
| BAI z-score | 16,370 | -0.07 (-0.11 to -0.04)^***^ | -0.03 (-0.06 to 0.004) | -0.02 (-0.06 to 0.008) |  |
| CMI z-score | 16,370 | -0.01 (-0.04 to 0.02) | 0.003 (-0.03 to 0.03) | 0.006 (-0.02 to 0.03) |  |
| BSA z-score | 16,370 | 0.02 (-0.02 to 0.05) | 0.006 (-0.03 to 0.04) | -0.004 (-0.04 to 0.03) |  |
| WHHR z-score | 16,370 | -0.15 (-0.19 to -0.12)^***^ | -0.09 (-0.12 to -0.06)^***^ | -0.07 (-0.09 to -0.04)^***^ |  |
| PFM z-score | 16,370 | -0.05 (-0.08 to -0.01)^**^ | -0.03 (-0.06 to 0.003) | -0.03 (-0.06 to 0.005) |  |
| PLM z-score | 16,370 | 0.05 (-0.01 to 0.10) | 0.02 (-0.03 to 0.07) | 0.003 (-0.05 to 0.05) |  |
| PPF z-score | 16,370 | -0.17 (-0.23 to -0.10)^***^ | -0.10 (-0.16 to -0.03)^**^ | -0.07 (-0.14 to -0.01)^*^ |  |
| CUN-BAE z-score | 16,370 | -0.09 (-0.13 to -0.04)^**^ | -0.05 (-0.10 to -0.002)^*^ | -0.04 (-0.09 to 0.003) |  |
| BMI groups |  |  |  |  |  |
| Underweight | 668 | 0.002 (-0.15 to 0.15) | 0.03 (-0.11 to 0.18) | 0.07 (-0.07 to 0.21) |  |
| Normal | 10,196 | Ref. (0) | Ref. (0) | Ref. (0) |  |
| Overweight | 3,471 | -0.05 (-0.12 to 0.03) | -0.01 (-0.08 to 0.06) | 0.001 (-0.07 to 0.07) |  |
| Obese | 2,035 | -0.14 (-0.23 to -0.05)^**^ | -0.07 (-0.16 to 0.02) | -0.05 (-0.14 to 0.03) |  |
| WC groups |  |  |  |  |  |
| Normal | 10,972 | Ref. (0) | Ref. (0) | Ref. (0) |  |
| High | 5,398 | -0.19 (-0.26 to -0.13)^***^ | -0.11 (-0.17 to -0.05)^**^ | -0.10 (-0.16 to -0.04)^**^ |  |
| BMI & WC groups |  |  |  |  |  |
| Non-obese | 9,373 | Ref. (0) | Ref. (0) | Ref. (0) |  |
| High BMI | 1,599 | 0.09 (-0.01 to 0.19) | 0.08 (-0.02 to 0.18) | 0.08 (-0.01 to 0.18) |  |
| High WC | 1,491 | -0.18 (-0.28 to -0.07)^**^ | -0.09 (-0.20 to 0.01) | -0.09 (-0.19 to 0.01) |  |
| High BMI & WC | 3,907 | -0.18 (-0.25 to -0.11)^***^ | -0.10 (-0.17 to -0.03)^**^ | -0.09 (-0.15 to -0.02)^*^ |  |
| WHR groups |  |  |  |  |  |
| Normal | 4,739 | Ref. (0) | Ref. (0) | Ref. (0) |  |
| High | 11,631 | -0.15 (-0.21 to -0.08)^***^ | -0.08 (-0.14 to -0.01)^*^ | -0.04 (-0.11 to 0.02) |  |
| WHtR groups |  |  |  |  |  |
| Normal | 8,155 | Ref. (0) | Ref. (0) | Ref. (0) |  |
| High | 8,215 | -0.20 (-0.25 to -0.14)^***^ | -0.11 (-0.17 to -0.05)^***^ | -0.09 (-0.14 to -0.03)^**^ |  |

ABSI: a body shape index; BAI: body adiposity index; BMI: body mass index; BRI: body roundness index; BSA: body surface area; CI: confidence interval; CMI: cardiometabolic index; CUN-BAE: Clínica Universidad de Navarra-Body Adiposity Estimator; CVAI: Chinese visceral adiposity index; DWRT: Delayed Word Recall Test; HC, hip circumference; LAP: lipid accumulation product; PFM: predicted fat mass; PLM: predicted lean mass; PPF: predicated percent fat; Ref, reference; VAI: visceral adiposity index; WC: waist circumference; WHHR: waist-to-hip-to-height ratio; WHR: waist-to-hip ratio; WHtR: waist-to-height ratio

^a^: Adjusted for sex and age

^b^: Additionally adjusted for education, occupation, personal income, physical activity, drinking, smoking and self-rated health

^c^: Additionally adjusted for baseline DWRT score

^d^: In model 3

^*^P <0.05, ^**^P <0.01, ^***^P <0.001

Underweight: BMI <18.5 kg/m^2^; Normal: 18.5 kg/m^2^ ≤ BMI <25 kg/m^2^; Overweight: 25 kg/m^2^ ≤ BMI < 27.5 kg/m^2^; Obese: BMI ≥ 27.5 kg/m^2^; High BMI: BMI ≥ 25 kg/m^2^

Normal WC: <90 cm for men, <80 cm for women; High WC: ≥90 cm for men, ≥80 cm for women

Normal WHR: <0.9 for men, <0.8 for women; High WHR: ≥0.9 for men, ≥0.8 for women

Normal WHtR: <0.5; High WHR: ≥0.5

Table S5. Associations of baseline obesity indicators with memory function at follow-up by education.

|  | *N* | Adjusted mean differences β (95% CI) in follow-up DWRT score | | |
| --- | --- | --- | --- | --- |
|  |  | Model 1^a^ | Model 2^b^ | Model 3^c^ |
| **Weight z-score** |  |  |  |  |
| Primary or less | 6,325 | -0.004 (-0.06 to 0.05) | -0.02 (-0.07 to 0.04) | -0.03 (-0.08 to 0.02) |
| Secondary or more | 10,045 | 0.006 (-0.03 to 0.04) | -0.003 (-0.04 to 0.04) | -0.003 (-0.04 to 0.03) |
| P for interaction |  | 0.32 | 0.51 | 0.93 |
| **BMI z-score** |  |  |  |  |
| Primary or less | 6,325 | -0.04 (-0.08 to 0.01) | -0.04 (-0.09 to 0.004) | -0.04 (-0.09 to 0.004) |
| Secondary or more | 10,045 | -0.02 (-0.06 to 0.02) | -0.02 (-0.06 to 0.01) | -0.02 (-0.05 to 0.02) |
| P for interaction |  | 0.63 | 0.57 | 0.52 |
| **WC z-score** |  |  |  |  |
| Primary or less | 6,325 | -0.06 (-0.12 to -0.01)^*^ | -0.06 (-0.11 to -0.01)^*^ | -0.06 (-0.11 to -0.01)^*^ |
| Secondary or more | 10,045 | -0.03 (-0.07 to 0.003) | -0.04 (-0.07 to 0.002) | -0.03 (-0.06 to 0.01) |
| P for interaction |  | 0.36 | 0.30 | 0.18 |
| **HC z-score** |  |  |  |  |
| Primary or less | 6,325 | 0.009 (-0.04 to 0.06) | 0.006 (-0.04 to 0.05) | -0.01 (-0.06 to 0.03) |
| Secondary or more | 10,045 | 0.009 (-0.03 to 0.04) | 0.001 (-0.03 to 0.04) | -0.0004 (-0.04 to 0.03) |
| P for interaction |  | 0.99 | 0.93 | 0.70 |
| **WHR z-score** |  |  |  |  |
| Primary or less | 6,325 | -0.11 (-0.16 to -0.06)^***^ | -0.11 (-0.16 to -0.05)^***^ | -0.08 (-0.13 to -0.03)^**^ |
| Secondary or more | 10,045 | -0.07 (-0.11 to -0.03)^**^ | -0.06 (-0.10 to -0.02)^**^ | -0.04 (-0.08 to -0.006)^*^ |
| P for interaction |  | 0.20 | 0.12 | 0.12 |
| **WHtR z-score** |  |  |  |  |
| Primary or less | 6,325 | -0.09 (-0.14 to -0.04)^**^ | -0.08 (-0.13 to -0.03)^**^ | -0.07 (-0.11 to -0.02)^**^ |
| Secondary or more | 10,045 | -0.05 (-0.09 to -0.01)^**^ | -0.05 (-0.09 to -0.01)^*^ | -0.04 (-0.07 to 0.001) |
| P for interaction |  | 0.05 | 0.06 | 0.06 |
| **LAP z-score** |  |  |  |  |
| Primary or less | 6,268 | -0.04 (-0.09 to 0.01) | -0.04 (-0.09 to 0.01) | -0.04 (-0.09 to 0.01) |
| Secondary or more | 9,927 | -0.003 (-0.04 to 0.04) | 0.002 (-0.04 to 0.04) | 0.005 (-0.03 to 0.04) |
| P for interaction |  | 0.16 | 0.17 | 0.13 |
| **ABSI z-score** |  |  |  |  |
| Primary or less | 6,325 | -0.08 (-0.14 to -0.03)^**^ | -0.07 (-0.12 to -0.01)^*^ | -0.05 (-0.10 to 0.004) |
| Secondary or more | 10,045 | -0.05 (-0.09 to -0.01)^*^ | -0.04 (-0.08 to -0.004)^*^ | -0.03 (-0.07 to 0.01) |
| P for interaction |  | 0.08 | 0.09 | 0.07 |
| **VAI z-score** |  |  |  |  |
| Primary or less | 6,325 | -0.02 (-0.07 to 0.03) | -0.02 (-0.07 to 0.03) | -0.02 (-0.07 to 0.03) |
| Secondary or more | 10,045 | 0.01 (-0.03 to 0.05) | 0.01 (-0.02 to 0.05) | 0.01 (-0.02 to 0.05) |
| P for interaction |  | 0.32 | 0.32 | 0.34 |
| **CVAI z-score** |  |  |  |  |
| Primary or less | 6,325 | -0.06 (-0.11 to -0.002)^*^ | -0.06 (-0.11 to -0.006)^*^ | -0.06 (-0.11 to -0.008)^*^ |
| Secondary or more | 10,045 | 0.005 (-0.03 to 0.04) | 0.005 (-0.03 to 0.04) | 0.008 (-0.03 to 0.04) |
| P for interaction |  | 0.002 | 0.003 | 0.001 |
| **BRI z-score** |  |  |  |  |
| Primary or less | 6,325 | -0.09 (-0.14 to -0.04)^***^ | -0.08 (-0.13 to -0.03)^**^ | -0.07 (-0.11 to -0.02)^**^ |
| Secondary or more | 10,045 | -0.05 (-0.09 to -0.01)^**^ | -0.05 (-0.09 to -0.01)^*^ | -0.04 (-0.07 to 0.001) |
| P for interaction |  | 0.05 | 0.06 | 0.06 |
| **Conicity index z-score** |  |  |  |  |
| Primary or less | 6,325 | -0.07 (-0.12 to -0.02)^**^ | -0.05 (-0.10 to 0.002) | -0.02 (-0.07 to 0.03) |
| Secondary or more | 10,045 | -0.05 (-0.09 to -0.007)^*^ | -0.03 (-0.07 to 0.01) | -0.02 (-0.06 to 0.02) |
| P for interaction |  | 0.02 | 0.03 | 0.08 |
| **BAI z-score** |  |  |  |  |
| Primary or less | 6,325 | -0.04 (-0.10 to 0.01) | -0.04 (-0.09 to 0.02) | -0.03 (-0.09 to 0.02) |
| Secondary or more | 10,045 | -0.03 (-0.07 to 0.02) | -0.03 (-0.07 to 0.02) | -0.02 (-0.06 to 0.02) |
| P for interaction |  | 0.047 | 0.10 | 0.18 |
| **CMI z-score** |  |  |  |  |
| Primary or less | 6,325 | -0.02 (-0.07 to 0.03) | -0.02 (-0.07 to 0.03) | -0.02 (-0.07 to 0.03) |
| Secondary or more | 10,045 | 0.01 (-0.02 to 0.05) | 0.02 (-0.02 to 0.05) | 0.02 (-0.02 to 0.05) |
| P for interaction |  | 0.43 | 0.38 | 0.37 |
| **BSA z-score** |  |  |  |  |
| Primary or less | 6,325 | 0.02 (-0.04 to 0.08) | 0.008 (-0.05 to 0.07) | -0.02 (-0.07 to 0.04) |
| Secondary or more | 10,045 | 0.02 (-0.02 to 0.06) | 0.009 (-0.03 to 0.05) | 0.005 (-0.03 to 0.05) |
| P for interaction |  | 0.07 | 0.16 | 0.54 |
| **WHHR z-score** |  |  |  |  |
| Primary or less | 6,325 | -0.13 (-0.18 to -0.08)^***^ | -0.12 (-0.18 to -0.07)^***^ | -0.08 (-0.13 to -0.03)^**^ |
| Secondary or more | 10,045 | -0.08 (-0.12 to -0.04)^***^ | -0.07 (-0.11 to -0.03)^***^ | -0.05 (-0.09 to -0.01)^**^ |
| P for interaction |  | 0.008 | 0.007 | 0.03 |
| **PFM z-score** |  |  |  |  |
| Primary or less | 6,325 | -0.03 (-0.09 to 0.03) | -0.04 (-0.10 to 0.02) | -0.05 (-0.10 to 0.009) |
| Secondary or more | 10,045 | -0.02 (-0.06 to 0.03) | -0.02 (-0.06 to 0.02) | -0.02 (-0.06 to 0.02) |
| P for interaction |  | 0.16 | 0.27 | 0.32 |
| **PLM z-score** |  |  |  |  |
| Primary or less | 6,325 | 0.05 (-0.05 to 0.14) | 0.02 (-0.07 to 0.12) | -0.02 (-0.11 to 0.07) |
| Secondary or more | 10,045 | 0.05 (-0.01 to 0.11) | 0.03 (-0.03 to 0.09) | 0.02 (-0.04 to 0.08) |
| P for interaction |  | 0.009 | 0.06 | 0.30 |
| **PPF z-score** |  |  |  |  |
| Primary or less | 6,325 | -0.13 (-0.25 to -0.01)^*^ | -0.14 (-0.26 to -0.03)^*^ | -0.12 (-0.23 to -0.01)^*^ |
| Secondary or more | 10,045 | -0.07 (-0.15 to 0.02) | -0.07 (-0.15 to 0.01) | -0.04 (-0.12 to 0.03) |
| P for interaction |  | 0.006 | 0.03 | 0.12 |
| **CUN-BAE z-score** |  |  |  |  |
| Primary or less | 6,325 | -0.06 (-0.14 to 0.02) | -0.07 (-0.15 to 0.01) | -0.07 (-0.15 to 0.01) |
| Secondary or more | 10,045 | -0.03 (-0.09 to 0.03) | -0.04 (-0.10 to 0.02) | -0.03 (-0.08 to 0.03) |
| P for interaction |  | 0.008 | 0.04 | 0.11 |

ABSI: a body shape index; BAI: body adiposity index; BMI: body mass index; BRI: body roundness index; BSA: body surface area; CI: confidence interval; CMI: cardiometabolic index; CUN-BAE: Clínica Universidad de Navarra-Body Adiposity Estimator; CVAI: Chinese visceral adiposity index; DWRT: Delayed Word Recall Test; HC, hip circumference; LAP: lipid accumulation product; PFM: predicted fat mass; PLM: predicted lean mass; PPF: predicated percent fat; Ref, reference; VAI: visceral adiposity index; WC: waist circumference; WHHR: waist-to-hip-to-height ratio; WHR: waist-to-hip ratio; WHtR: waist-to-height ratio

^a^: Adjusted for sex and age

^b^: Additionally adjusted for occupation, personal income, physical activity, drinking, smoking and self-rated health

^c^: Additionally adjusted for baseline DWRT score

^*^P <0.05, ^**^P <0.01, ^***^P <0.001

Underweight: BMI <18.5 kg/m^2^; Normal: 18.5 kg/m^2^ ≤ BMI <25 kg/m^2^; Overweight: 25 kg/m^2^ ≤ BMI < 27.5 kg/m^2^; Obese: BMI ≥ 27.5 kg/m^2^; High BMI: BMI ≥ 25 kg/m^2^

Normal WC: <90 cm for men, <80 cm for women; High WC: ≥90 cm for men, ≥80 cm for women

Normal WHR: <0.9 for men, <0.8 for women; High WHR: ≥0.9 for men, ≥0.8 for women

Normal WHtR: <0.5; High WHR: ≥0.5

Table S6. Associations of baseline obesity indicators with memory impairment at follow-up in participants without memory impairment at baseline.

|  | *N* (% cases) | Person years | Adjusted HR (95% CI) of memory impairment at follow-up (DWRT < 4) | | |
| --- | --- | --- | --- | --- | --- |
|  |  |  | Model 1^a^ | Model 2^b^ | Model 3^c^ |
| Weight, kg | 14,613 (7.01) | 57,058 | 1.00 (0.99 to 1.00) | 1.00 (0.99 to 1.00) | 1.00 (0.99 to 1.00) |
| BMI, kg/m^2^ | 14,613 (7.01) | 57,058 | 1.01 (0.99 to 1.03) | 1.00 (0.98 to 1.02) | 1.00 (0.98 to 1.02) |
| WC, cm | 14,613 (7.01) | 57,058 | 1.01 (1.00 to 1.02)^**^ | 1.01 (1.00 to 1.01) | 1.01 (1.00 to 1.01) |
| HC, cm | 14,613 (7.01) | 57,058 | 1.00 (0.99 to 1.01) | 1.00 (0.99 to 1.01) | 1.00 (0.99 to 1.01) |
| WHR | 14,613 (7.01) | 57,058 | 10.96 (4.16 to 28.69)^***^ | 5.32 (2.02 to 14.00)^**^ | 4.39 (1.66 to 11.63)^**^ |
| WHtR | 14,613 (7.01) | 57,058 | 10.07 (3.33 to 30.46)^***^ | 4.07 (1.34 to 12.36)^*^ | 3.50 (1.16 to 10.58)^*^ |
| LAP | 14,456 (6.99) | 56,452 | 1.00 (1.00 to 1.00) | 1.00 (1.00 to 1.00) | 1.00 (1.00 to 1.00) |
| ABSI | 14,613 (7.01) | 57,058 | 2.74e15 (4.31e09 to 1.75e21)^***^ | 1.87e11 (2.39e05 to 1.46e17)^***^ | 1.36e10 (1.71e04 to 1.08e16)^**^ |
| VAI | 14,613 (7.01) | 57,058 | 0.99 (0.95 to 1.03) | 0.98 (0.94 to 1.02) | 0.98 (0.94 to 1.02) |
| CVAI | 14,613 (7.01) | 57,058 | 1.00 (1.00 to 1.00) | 1.00 (1.00 to 1.00) | 1.00 (1.00 to 1.00) |
| BRI | 14,613 (7.01) | 57,058 | 1.13 (1.07 to 1.19)^***^ | 1.08 (1.02 to 1.14)^*^ | 1.07 (1.01 to 1.13)^*^ |
| Conicity index | 14,613 (7.01) | 57,058 | 1.40e05 (1.39e03 to 1.42e07)^***^ | 9.19e03 (83.27 to 1.01e006)^***^ | 4.04e03 (35.50 to 4.61e05)^**^ |
| BAI | 14,613 (7.01) | 57,058 | 1.02 (1.00 to 1.04)^*^ | 1.01 (0.99 to 1.03) | 1.01 (0.99 to 1.03) |
| CMI | 14,613 (7.01) | 57,058 | 0.95 (0.84 to 1.07) | 0.93 (0.82 to 1.05) | 0.93 (0.83 to 1.06) |
| BSA | 14,613 (7.01) | 57,058 | 0.57 (0.35 to 0.93)^*^ | 0.64 (0.39 to 1.04) | 0.67 (0.41 to 1.09) |
| WHHR | 14,613 (7.01) | 57,058 | 9.8e182 (4.5e124 to 2.2e241)^***^ | 8.3e126 (6.15e67 to 1.1e186)^***^ | 1.1e114 (2.53e54 to 4.8e173)^***^ |
| PFM | 14,613 (7.01) | 57,058 | 1.00 (0.99 to 1.01) | 1.00 (0.99 to 1.01) | 1.00 (0.99 to 1.01) |
| PLM | 14,613 (7.01) | 57,058 | 0.98 (0.96 to 0.99)^**^ | 0.98 (0.97 to 1.00)^*^ | 0.99 (0.97 to 1.00) |
| PPF | 14,613 (7.01) | 57,058 | 1.02 (1.00 to 1.04) | 1.01 (0.99 to 1.03) | 1.01 (0.99 to 1.03) |
| CUN-BAE | 14,613 (7.01) | 57,058 | 1.00 (0.99 to 1.02) | 1.00 (0.98 to 1.01) | 1.00 (0.98 to 1.01) |
| Weight z-score | 14,613 (7.01) | 57,058 | 0.96 (0.90 to 1.02) | 0.96 (0.90 to 1.03) | 0.96 (0.90 to 1.03) |
| BMI z-score | 14,613 (7.01) | 57,058 | 1.02 (0.96 to 1.09) | 1.00 (0.94 to 1.06) | 1.00 (0.94 to 1.06) |
| WC z-score | 14,613 (7.01) | 57,058 | 1.09 (1.03 to 1.16)^**^ | 1.05 (0.99 to 1.12) | 1.05 (0.98 to 1.12) |
| HC z-score | 14,613 (7.01) | 57,058 | 0.98 (0.92 to 1.04) | 0.97 (0.91 to 1.03) | 0.98 (0.92 to 1.04) |
| WHR z-score | 14,613 (7.01) | 57,058 | 1.18 (1.10 to 1.26)^***^ | 1.12 (1.05 to 1.20)^**^ | 1.11 (1.03 to 1.18)^**^ |
| WHtR z-score | 14,613 (7.01) | 57,058 | 1.14 (1.07 to 1.22)^***^ | 1.08 (1.02 to 1.15)^*^ | 1.07 (1.01 to 1.14)^*^ |
| LAP z-score | 14,456 (6.99) | 56,452 | 1.04 (0.98 to 1.11) | 1.02 (0.95 to 1.09) | 1.02 (0.96 to 1.09) |
| ABSI z-score | 14,613 (7.01) | 57,058 | 1.19 (1.12 to 1.27)^***^ | 1.14 (1.06 to 1.21)^***^ | 1.12 (1.05 to 1.20)^**^ |
| VAI z-score | 14,613 (7.01) | 57,058 | 0.97 (0.91 to 1.05) | 0.96 (0.89 to 1.04) | 0.97 (0.90 to 1.04) |
| CVAI z-score | 14,613 (7.01) | 57,058 | 1.02 (0.96 to 1.09) | 1.00 (0.94 to 1.07) | 1.00 (0.94 to 1.07) |
| BRI z-score | 14,613 (7.01) | 57,058 | 1.14 (1.07 to 1.22)^***^ | 1.09 (1.02 to 1.15)^*^ | 1.08 (1.01 to 1.14)^*^ |
| Conicity index z-score | 14,613 (7.01) | 57,058 | 1.18 (1.11 to 1.26)^***^ | 1.14 (1.06 to 1.22)^***^ | 1.13 (1.05 to 1.20)^**^ |
| BAI z-score | 14,613 (7.01) | 57,058 | 1.08 (1.00 to 1.16)^*^ | 1.03 (0.96 to 1.11) | 1.03 (0.96 to 1.11) |
| CMI z-score | 14,613 (7.01) | 57,058 | 0.97 (0.90 to 1.04) | 0.96 (0.89 to 1.03) | 0.96 (0.89 to 1.03) |
| BSA z-score | 14,613 (7.01) | 57,058 | 0.92 (0.86 to 0.99)^*^ | 0.94 (0.87 to 1.01) | 0.94 (0.88 to 1.01) |
| WHHR z-score | 14,613 (7.01) | 57,058 | 1.22 (1.14 to 1.30)^***^ | 1.15 (1.08 to 1.22)^***^ | 1.13 (1.06 to 1.21)^***^ |
| PFM z-score | 14,613 (7.01) | 57,058 | 1.00 (0.93 to 1.08) | 0.99 (0.92 to 1.06) | 0.99 (0.92 to 1.06) |
| PLM z-score | 14,613 (7.01) | 57,058 | 0.85 (0.76 to 0.96)^**^ | 0.88 (0.79 to 0.99)^*^ | 0.89 (0.80 to 1.00) |
| PPF z-score | 14,613 (7.01) | 57,058 | 1.14 (0.99 to 1.31) | 1.07 (0.93 to 1.23) | 1.06 (0.92 to 1.21) |
| CUN-BAE z-score | 14,613 (7.01) | 57,058 | 1.03 (0.93 to 1.15) | 1.00 (0.90 to 1.11) | 1.00 (0.90 to 1.10) |
| BMI groups |  |  |  |  |  |
| Underweight | 580 (7.93) | 2,277 | 1.03 (0.77 to 1.40) | 1.02 (0.76 to 1.38) | 0.99 (0.73 to 1.34) |
| Normal | 9,142 (6.91) | 35,694 | Ref. (1) | Ref. (1) | Ref. (1) |
| Overweight | 3,084 (7.23) | 12,025 | 1.04 (0.89 to 1.21) | 1.01 (0.86 to 1.17) | 1.00 (0.86 to 1.17) |
| Obese | 1,807 (6.81) | 7,062 | 1.00 (0.82 to 1.21) | 0.95 (0.78 to 1.15) | 0.94 (0.77 to 1.14) |
| WC groups |  |  |  |  |  |
| Normal | 9,839 (6.31) | 38,272 | Ref. (1) | Ref. (1) | Ref. (1) |
| High | 4,774 (8.44) | 18,785 | 1.29 (1.13 to 1.47)^***^ | 1.18 (1.04 to 1.35)^*^ | 1.16 (1.02 to 1.33)^*^ |
| BMI & WC groups |  |  |  |  |  |
| Non-obese | 8,401 (6.56) | 32,687 | Ref. (1) | Ref. (1) | Ref. (1) |
| High BMI | 1,438 (4.87) | 5,585 | 0.76 (0.59 to 0.97)^*^ | 0.78 (0.61 to 1.00) | 0.79 (0.61 to 1.01) |
| High WC | 1,321 (9.61) | 5,283 | 1.36 (1.11 to 1.66)^**^ | 1.25 (1.02 to 1.53)^*^ | 1.23 (1.00 to 1.50)^*^ |
| High BMI & WC | 3,453 (7.99) | 13,502 | 1.21 (1.04 to 1.40)^*^ | 1.11 (0.96 to 1.29) | 1.10 (0.95 to 1.27) |
| WHR groups |  |  |  |  |  |
| Normal | 4,306 (6.29) | 16,809 | Ref. (1) | Ref. (1) | Ref. (1) |
| High | 10,307 (7.31) | 40,249 | 1.18 (1.02 to 1.37)^*^ | 1.11 (0.95 to 1.28) | 1.08 (0.93 to 1.25) |
| WHtR groups |  |  |  |  |  |
| Normal | 7,400 (5.76) | 28,665 | Ref. (1) | Ref. (1) | Ref. (1) |
| High | 7,213 (8.29) | 28,392 | 1.23 (1.08 to 1.39)^**^ | 1.14 (1.00 to 1.29) | 1.11 (0.98 to 1.26) |

ABSI: a body shape index; BAI: body adiposity index; BMI: body mass index; BRI: body roundness index; BSA: body surface area; CI: confidence interval; CMI: cardiometabolic index; CUN-BAE: Clínica Universidad de Navarra-Body Adiposity Estimator; CVAI: Chinese visceral adiposity index; DWRT: Delayed Word Recall Test; HC, hip circumference; HR, hazard ratio; LAP: lipid accumulation product; PFM: predicted fat mass; PLM: predicted lean mass; PPF: predicated percent fat; Ref, reference; VAI: visceral adiposity index; WC: waist circumference; WHHR: waist-to-hip-to-height ratio; WHR: waist-to-hip ratio; WHtR: waist-to-height ratio

^a^: Adjusted for sex and age

^b^: Additionally adjusted for education, occupation, personal income, physical activity, drinking, smoking and self-rated health

^c^: Additionally adjusted for baseline DWRT score

^*^P <0.05, ^**^P <0.01, ^***^P <0.001

Underweight: BMI <18.5 kg/m^2^; Normal: 18.5 kg/m^2^ ≤ BMI <25 kg/m^2^; Overweight: 25 kg/m^2^ ≤ BMI < 27.5 kg/m^2^; Obese: BMI ≥ 27.5 kg/m^2^; High BMI: BMI ≥ 25 kg/m^2^

Normal WC: <90 cm for men, <80 cm for women; High WC: ≥90 cm for men, ≥80 cm for women

Normal WHR: <0.9 for men, <0.8 for women; High WHR: ≥0.9 for men, ≥0.8 for women

Normal WHtR: <0.5; High WHR: ≥0.5

Table S7. Associations of obesity indicators with memory function using generalised estimating equation.

|  | Adjusted mean differences β (95% CI) in DWRT score | | |
| --- | --- | --- | --- |
|  | Model 1^a^ | Model 2^b^ | Model 3^c^ |
| Weight, kg | 0.003 (0.001 to 0.005)^**^ | 0.004 (0.002 to 0.006)^***^ | 0.002 (0.0004 to 0.004)^*^ |
| BMI, kg/m^2^ | -0.002 (-0.008 to 0.004) | -0.007 (-0.01 to -0.001)^*^ | -0.001 (-0.007 to 0.004) |
| WC, cm | -0.01 (-0.01 to -0.009)^***^ | -0.002 (-0.004 to 0.0002) | -0.002 (-0.004 to 0.0001) |
| HC, cm | 0.006 (0.003 to 0.009)^***^ | 0.006 (0.003 to 0.01)^***^ | 0.007 (0.004 to 0.01)^***^ |
| WHR | -2.90 (-3.17 to -2.63)^***^ | -1.30 (-1.59 to -1.02)^***^ | -0.80 (-1.07 to -0.51)^***^ |
| WHtR | -2.22 (-2.54 to -1.90)^***^ | -1.00 (-1.32 to -0.68)^***^ | -0.64 (-0.95 to -0.32)^***^ |
| LAP | -0.001 (-0.002 to -0.0006)^***^ | -0.0008 (-0.001 to -0.0003)^**^ | -0.0005 (-0.001 to -5.48e-06)^*^ |
| ABSI | -36.02 (-39.55 to -32.48)^***^ | -7.01 (-10.70 to -3.33)^***^ | -8.62 (-12.27 to -4.97)^***^ |
| VAI | 0.007 (-0.002 to 0.02) | 0.002 (-0.007 to 0.01) | -0.002 (-0.01 to 0.007) |
| CVAI | -0.002 (-0.003 to -0.002)^***^ | -0.0001 (-0.0006 to 0.0003) | -0.00007 (-0.0005 to 0.0004) |
| BRI | -0.12 (-0.13 to -0.10)^***^ | -0.05 (-0.07 to -0.04)^***^ | -0.03 (-0.05 to -0.02)^***^ |
| Conicity index | -11.18 (-12.49 to -9.88)^***^ | -4.69 (-6.06 to -3.32)^***^ | -3.69 (-5.03 to -2.36)^***^ |
| BAI | -0.003 (-0.008 to 0.001) | -0.008 (-0.01 to -0.003)^**^ | -0.004 (-0.009 to 0.001) |
| CMI | -0.01 (-0.04 to 0.02) | 0.003 (-0.02 to 0.03) | -0.004 (-0.03 to 0.02) |
| BSA | 0.34 (0.21 to 0.47)^***^ | 0.56 (0.41 to 0.71)^***^ | 0.29 (0.14 to 0.43)^***^ |
| WHHR | -463.65 (-503.21 to -424.09)^***^ | -288.04 (-328.29 to -247.80)^***^ | -160.14 (-199.57 to -120.71)^***^ |
| PFM | 0.01 (0.009 to 0.02)^***^ | 0.001 (-0.002 to 0.005) | 0.0006 (-0.003 to 0.004) |
| PLM | -0.003 (-0.005 to -0.00001)^*^ | 0.02 (0.01 to 0.02)^***^ | 0.01 (0.006 to 0.02)^***^ |
| PPF | 0.01 (0.008 to 0.01)^***^ | -0.01 (-0.02 to -0.004)^**^ | -0.006 (-0.01 to -0.0001)^*^ |
| CUN-BAE | 0.007 (0.004 to 0.009)^***^ | -0.005 (-0.009 to -0.0005)^*^ | -0.001 (-0.006 to 0.003) |
| Weight z-score | 0.03 (0.01 to 0.05)^**^ | 0.03 (0.01 to 0.05)^**^ | 0.02 (0.001 to 0.04)^*^ |
| BMI z-score | -0.01 (-0.03 to 0.01) | -0.03 (-0.04 to -0.007)^**^ | -0.01 (-0.02 to 0.01) |
| WC z-score | -0.15 (-0.17 to -0.13)^***^ | -0.08 (-0.10 to -0.06)^***^ | -0.04 (-0.06 to -0.02)^***^ |
| HC z-score | 0.01 (-0.01 to 0.03) | 0.003 (-0.02 to 0.02) | 0.007 (-0.01 to 0.03) |
| WHR z-score | -0.23 (-0.24 to -0.21)^***^ | -0.13 (-0.15 to -0.11)^***^ | -0.07 (-0.09 to -0.05)^***^ |
| WHtR z-score | -0.18 (-0.20 to -0.16)^***^ | -0.12 (-0.14 to -0.10)^***^ | -0.06 (-0.08 to -0.04)^***^ |
| LAP z-score | -0.06 (-0.08 to -0.04)^***^ | -0.06 (-0.08 to -0.04)^***^ | -0.03 (-0.05 to -0.01)^**^ |
| ABSI z-score | -0.28 (-0.29 to -0.26)^***^ | -0.16 (-0.17 to -0.14)^***^ | -0.09 (-0.11 to -0.07)^***^ |
| VAI z-score | -0.02 (-0.04 to 0.0005) | -0.04 (-0.06 to -0.02)^***^ | -0.02 (-0.04 to -0.001)^*^ |
| CVAI z-score | -0.15 (-0.17 to -0.13)^***^ | -0.07 (-0.09 to -0.05)^***^ | -0.03 (-0.05 to -0.008)^**^ |
| BRI z-score | -0.18 (-0.20 to -0.16)^***^ | -0.13 (-0.14 to -0.11)^***^ | -0.06 (-0.08 to -0.04)^***^ |
| Conicity index z-score | -0.23 (-0.25 to -0.21)^***^ | -0.15 (-0.17 to -0.13)^***^ | -0.09 (-0.10 to -0.07)^***^ |
| BAI z-score | -0.05 (-0.07 to -0.03)^***^ | -0.09 (-0.11 to -0.07)^***^ | -0.04 (-0.06 to -0.02)^**^ |
| CMI z-score | -0.04 (-0.06 to -0.02)^***^ | -0.04 (-0.05 to -0.02)^***^ | -0.02 (-0.03 to 0.001) |
| BSA z-score | 0.05 (0.03 to 0.07)^***^ | 0.07 (0.05 to 0.10)^***^ | 0.04 (0.02 to 0.06)^***^ |
| WHHR z-score | -0.24 (-0.26 to -0.23)^***^ | -0.17 (-0.19 to -0.15)^***^ | -0.09 (-0.11 to -0.07)^***^ |
| PFM z-score | 0.06 (0.04 to 0.08)^***^ | -0.02 (-0.04 to 0.004) | -0.005 (-0.03 to 0.02) |
| PLM z-score | -0.01 (-0.03 to 0.005) | 0.15 (0.12 to 0.19)^***^ | 0.09 (0.05 to 0.12)^***^ |
| PPF z-score | 0.06 (0.04 to 0.08)^***^ | -0.17 (-0.22 to -0.13)^***^ | -0.08 (-0.12 to -0.04)^***^ |
| CUN-BAE z-score | 0.03 (0.01 to 0.05)^**^ | -0.08 (-0.11 to -0.04)^***^ | -0.02 (-0.05 to 0.01) |
| BMI groups |  |  |  |
| Underweight | -0.17 (-0.26 to -0.07)^***^ | -0.08 (-0.17 to 0.01) | -0.03 (-0.12 to 0.06) |
| Normal | Ref. (0) | Ref. (0) | Ref. (0) |
| Overweight | -0.04 (-0.08 to 0.01) | -0.03 (-0.08 to 0.01) | -0.003 (-0.05 to 0.04) |
| Obese | -0.07 (-0.13 to -0.02)^*^ | -0.09 (-0.14 to -0.03)^**^ | -0.02 (-0.07 to 0.04) |
| WC groups |  |  |  |
| Normal | Ref. (0) | Ref. (0) | Ref. (0) |
| High | -0.09 (-0.13 to -0.05)^***^ | -0.05 (-0.09 to -0.01)^**^ | -0.03 (-0.06 to 0.01) |
| BMI & WC groups |  |  |  |
| Non-obese | Ref. (0) | Ref. (0) | Ref. (0) |
| High BMI | 0.04 (-0.03 to 0.11) | -0.002 (-0.07 to 0.06) | 0.02 (-0.05 to 0.09) |
| High WC | -0.09 (-0.15 to -0.04)^**^ | -0.02 (-0.07 to 0.04) | -0.03 (-0.09 to 0.02) |
| High BMI & WC | -0.08 (-0.13 to -0.04)^***^ | -0.07 (-0.11 to -0.02)^**^ | -0.02 (-0.06 to 0.02) |
| WHR groups |  |  |  |
| Normal | Ref. (0) | Ref. (0) | Ref. (0) |
| High | -0.09 (-0.13 to -0.05)^***^ | -0.10 (-0.14 to -0.05)^***^ | -0.06 (-0.10 to -0.02)^**^ |
| WHtR groups |  |  |  |
| Normal | Ref. (0) | Ref. (0) | Ref. (0) |
| High | -0.21 (-0.25 to -0.17)^***^ | -0.08 (-0.12 to -0.04)^***^ | -0.05 (-0.09 to -0.01)^**^ |

ABSI: a body shape index; BAI: body adiposity index; BMI: body mass index; BRI: body roundness index; BSA: body surface area; CI: confidence interval; CMI: cardiometabolic index; CUN-BAE: Clínica Universidad de Navarra-Body Adiposity Estimator; CVAI: Chinese visceral adiposity index; DWRT: Delayed Word Recall Test; HC, hip circumference; LAP: lipid accumulation product; PFM: predicted fat mass; PLM: predicted lean mass; PPF: predicated percent fat; Ref, reference; VAI: visceral adiposity index; WC: waist circumference; WHHR: waist-to-hip-to-height ratio; WHR: waist-to-hip ratio; WHtR: waist-to-height ratio

^a^: Unadjusted

^b^: Adjusted for sex and age

^c^: Additionally adjusted for education, occupation, personal income, physical activity, drinking, smoking and self-rated health

^*^P <0.05, ^**^P <0.01, ^***^P <0.001

Underweight: BMI <18.5 kg/m^2^; Normal: 18.5 kg/m^2^ ≤ BMI <25 kg/m^2^; Overweight: 25 kg/m^2^ ≤ BMI < 27.5 kg/m^2^; Obese: BMI ≥ 27.5 kg/m^2^; High BMI: BMI ≥ 25 kg/m^2^

Normal WC: <90 cm for men, <80 cm for women; High WC: ≥90 cm for men, ≥80 cm for women

Normal WHR: <0.9 for men, <0.8 for women; High WHR: ≥0.9 for men, ≥0.8 for women

Normal WHtR: <0.5; High WHR: ≥0.5

Table S8. Associations of baseline obesity indicators with mean annual change of memory function.

|  | *N* | Adjusted mean differences β (95% CI) in mean annual change of DWRT score | | |
| --- | --- | --- | --- | --- |
|  |  | Model 1^a^ | Model 2^b^ | Model 3^c^ |
| Weight, kg | 16,370 | -0.0007 (-0.002 to 0.0003) | -0.0006 (-0.002 to 0.0004) | -0.0004 (-0.001 to 0.0005) |
| BMI, kg/m^2^ | 16,370 | -0.001 (-0.004 to 0.001) | -0.001 (-0.004 to 0.001) | -0.002 (-0.004 to 0.00002) |
| WC, cm | 16,370 | -0.0006 (-0.002 to 0.0004) | -0.0006 (-0.002 to 0.0005) | -0.001 (-0.002 to -0.0004)^**^ |
| HC, cm | 16,370 | -0.0007 (-0.002 to 0.0006) | -0.0007 (-0.002 to 0.0007) | -0.00003 (-0.001 to 0.001) |
| WHR | 16,370 | -0.07 (-0.21 to 0.07) | -0.06 (-0.20 to 0.08) | -0.25 (-0.37 to -0.14)^***^ |
| WHtR | 16,370 | -0.08 (-0.24 to 0.08) | -0.08 (-0.24 to 0.08) | -0.24 (-0.37 to -0.10)^**^ |
| LAP | 16,195 | -0.0001 (-0.0004 to 0.0002) | -0.00009 (-0.0004 to 0.0002) | -0.0001 (-0.0004 to 0.00008) |
| ABSI | 16,370 | -0.14 (-2.07 to 1.80) | -0.14 (-2.09 to 1.82) | -2.25 (-3.88 to -0.62)^**^ |
| VAI | 16,370 | -0.0001 (-0.005 to 0.005) | 0.0002 (-0.005 to 0.005) | -0.0004 (-0.004 to 0.004) |
| CVAI | 16,370 | -0.00009 (-0.0003 to 0.0001) | -0.00008 (-0.0003 to 0.0001) | -0.0001 (-0.0003 to 0.00006) |
| BRI | 16,370 | -0.004 (-0.01 to 0.004) | -0.004 (-0.01 to 0.004) | -0.01 (-0.02 to -0.006)^***^ |
| Conicity index | 16,370 | 0.34 (-0.34 to 1.03) | 0.29 (-0.40 to 0.98) | -0.48 (-1.05 to 0.09) |
| BAI | 16,370 | -0.0005 (-0.003 to 0.002) | -0.0006 (-0.003 to 0.002) | -0.001 (-0.003 to 0.0007) |
| CMI | 16,370 | 0.001 (-0.01 to 0.02) | 0.002 (-0.01 to 0.02) | 0.0002 (-0.01 to 0.01) |
| BSA | 16,370 | -0.05 (-0.12 to 0.02) | -0.05 (-0.12 to 0.02) | -0.006 (-0.06 to 0.05) |
| WHHR | 16,370 | -5.79 (-25.54 to 13.95) | -5.15 (-25.09 to 14.78) | -40.28 (-56.88 to -23.68)^***^ |
| PFM | 16,370 | -0.0009 (-0.003 to 0.0007) | -0.0008 (-0.002 to 0.0009) | -0.001 (-0.002 to 0.0003) |
| PLM | 16,370 | -0.002 (-0.004 to 0.0004) | -0.001 (-0.004 to 0.0007) | 0.0001 (-0.002 to 0.002) |
| PPF | 16,370 | -0.0008 (-0.004 to 0.002) | -0.0006 (-0.003 to 0.002) | -0.003 (-0.005 to -0.0003)^*^ |
| CUN-BAE | 16,370 | -0.001 (-0.003 to 0.001) | -0.001 (-0.003 to 0.001) | -0.002 (-0.003 to 0.0001) |
| Weight z-score | 16,370 | -0.007 (-0.02 to 0.003) | -0.006 (-0.02 to 0.004) | -0.003 (-0.01 to 0.004) |
| BMI z-score | 16,370 | -0.005 (-0.01 to 0.004) | -0.004 (-0.01 to 0.005) | -0.007 (-0.01 to 0.0001) |
| WC z-score | 16,370 | -0.006 (-0.01 to 0.003) | -0.005 (-0.01 to 0.004) | -0.01 (-0.02 to -0.003)^**^ |
| HC z-score | 16,370 | -0.005 (-0.01 to 0.004) | -0.005 (-0.01 to 0.004) | -0.0001 (-0.008 to 0.007) |
| WHR z-score | 16,370 | -0.005 (-0.01 to 0.005) | -0.004 (-0.01 to 0.006) | -0.02 (-0.03 to -0.01)^***^ |
| WHtR z-score | 16,370 | -0.005 (-0.01 to 0.004) | -0.004 (-0.01 to 0.005) | -0.01 (-0.02 to -0.006)^**^ |
| LAP z-score | 16,195 | -0.004 (-0.01 to 0.005) | -0.003 (-0.01 to 0.006) | -0.005 (-0.01 to 0.003) |
| ABSI z-score | 16,370 | -0.0007 (-0.01 to 0.009) | -0.0007 (-0.01 to 0.009) | -0.01 (-0.02 to -0.003)^**^ |
| VAI z-score | 16,370 | -0.0003 (-0.009 to 0.009) | 0.0004 (-0.008 to 0.009) | -0.0007 (-0.008 to 0.007) |
| CVAI z-score | 16,370 | -0.004 (-0.01 to 0.005) | -0.003 (-0.01 to 0.006) | -0.005 (-0.01 to 0.003) |
| BRI z-score | 16,370 | -0.005 (-0.01 to 0.004) | -0.004 (-0.01 to 0.005) | -0.01 (-0.02 to -0.006)^***^ |
| Conicity index z-score | 16,370 | 0.005 (-0.005 to 0.01) | 0.004 (-0.006 to 0.01) | -0.007 (-0.01 to 0.001) |
| BAI z-score | 16,370 | -0.002 (-0.01 to 0.008) | -0.003 (-0.01 to 0.008) | -0.006 (-0.01 to 0.003) |
| CMI z-score | 16,370 | 0.0008 (-0.008 to 0.01) | 0.001 (-0.007 to 0.01) | 0.0001 (-0.007 to 0.007) |
| BSA z-score | 16,370 | -0.008 (-0.02 to 0.002) | -0.007 (-0.02 to 0.004) | -0.0008 (-0.009 to 0.008) |
| WHHR z-score | 16,370 | -0.003 (-0.01 to 0.007) | -0.002 (-0.01 to 0.007) | -0.02 (-0.03 to -0.01)^***^ |
| PFM z-score | 16,370 | -0.006 (-0.02 to 0.005) | -0.005 (-0.02 to 0.006) | -0.007 (-0.02 to 0.002) |
| PLM z-score | 16,370 | -0.01 (-0.03 to 0.003) | -0.01 (-0.03 to 0.005) | 0.0008 (-0.01 to 0.01) |
| PPF z-score | 16,370 | -0.006 (-0.03 to 0.01) | -0.005 (-0.02 to 0.02) | -0.02 (-0.04 to -0.002)^*^ |
| CUN-BAE z-score | 16,370 | -0.008 (-0.02 to 0.007) | -0.007 (-0.02 to 0.008) | -0.01 (-0.02 to 0.0007) |
| BMI groups |  |  |  |  |
| Underweight | 668 | 0.04 (-0.0004 to 0.09) | 0.04 (-0.003 to 0.09) | 0.01 (-0.02 to 0.06) |
| Normal | 10,196 | Ref. (0) | Ref. (0) | Ref. (0) |
| Overweight | 3,471 | 0.008 (-0.01 to 0.03) | 0.008 (-0.01 to 0.03) | 0.002 (-0.02 to 0.02) |
| Obese | 2,035 | -0.008 (-0.04 to 0.02) | -0.008 (-0.03 to 0.02) | -0.02 (-0.04 to 0.007) |
| WC groups |  |  |  |  |
| Normal | 10,972 | Ref. (0) | Ref. (0) | Ref. (0) |
| High | 5,398 | -0.02 (-0.04 to -0.002)^*^ | -0.02 (-0.04 to -0.002)^*^ | -0.03 (-0.04 to -0.01)^**^ |
| BMI & WC groups |  |  |  |  |
| Non-obese | 9,373 | Ref. (0) | Ref. (0) | Ref. (0) |
| High BMI | 1,599 | 0.02 (-0.005 to 0.05) | 0.03 (-0.002 to 0.06) | 0.02 (-0.0004 to 0.05) |
| High WC | 1,491 | -0.02 (-0.05 to 0.01) | -0.02 (-0.05 to 0.01) | -0.02 (-0.05 to 0.001) |
| High BMI & WC | 3,907 | -0.02 (-0.04 to 0.01) | -0.02 (-0.04 to 0.006) | -0.02 (-0.04 to -0.006)^**^ |
| WHR groups |  |  |  |  |
| Normal | 4,739 | Ref. (0) | Ref. (0) | Ref. (0) |
| High | 11,631 | 0.01 (-0.01 to 0.03) | 0.01 (-0.01 to 0.03) | -0.01 (-0.03 to 0.005) |
| WHtR groups |  |  |  |  |
| Normal | 8,155 | Ref. (0) | Ref. (0) | Ref. (0) |
| High | 8,215 | -0.006 (-0.02 to 0.01) | -0.006 (-0.02 to 0.01) | -0.02 (-0.04 to -0.008)^**^ |

ABSI: a body shape index; BAI: body adiposity index; BMI: body mass index; BRI: body roundness index; BSA: body surface area; CI: confidence interval; CMI: cardiometabolic index; CUN-BAE: Clínica Universidad de Navarra-Body Adiposity Estimator; CVAI: Chinese visceral adiposity index; DWRT: Delayed Word Recall Test; HC, hip circumference; LAP: lipid accumulation product; PFM: predicted fat mass; PLM: predicted lean mass; PPF: predicated percent fat; Ref, reference; VAI: visceral adiposity index; WC: waist circumference; WHHR: waist-to-hip-to-height ratio; WHR: waist-to-hip ratio; WHtR: waist-to-height ratio

^a^: Adjusted for sex and age

^b^: Additionally adjusted for education, occupation, personal income, physical activity, drinking, smoking and self-rated health

^c^: Additionally adjusted for baseline DWRT score

^*^P <0.05, ^**^P <0.01, ^***^P <0.001

Underweight: BMI <18.5 kg/m^2^; Normal: 18.5 kg/m^2^ ≤ BMI <25 kg/m^2^; Overweight: 25 kg/m^2^ ≤ BMI < 27.5 kg/m^2^; Obese: BMI ≥ 27.5 kg/m^2^; High BMI: BMI ≥ 25 kg/m^2^

Normal WC: <90 cm for men, <80 cm for women; High WC: ≥90 cm for men, ≥80 cm for women

Normal WHR: <0.9 for men, <0.8 for women; High WHR: ≥0.9 for men, ≥0.8 for women

Normal WHtR: <0.5; High WHR: ≥0.5

Table S9. Associations of baseline obesity indicators with mean annual change of memory function by education.

|  | *N* | Adjusted mean differences β (95% CI) in mean annual change of DWRT score | | |
| --- | --- | --- | --- | --- |
|  |  | Model 1^a^ | Model 2^b^ | Model 3^c^ |
| **Weight z-score** |  |  |  |  |
| Primary or less | 6,325 | -0.01 (-0.03 to 0.0007) | -0.01 (-0.03 to 0.0007) | -0.007 (-0.02 to 0.006) |
| Secondary or more | 10,045 | -0.002 (-0.01 to 0.01) | -0.008 (-0.01 to 0.01) | -0.001 (-0.01 to 0.009) |
| P for interaction |  | 0.30 | 0.33 | 0.84 |
| **BMI z-score** |  |  |  |  |
| Primary or less | 6,325 | -0.009 (-0.02 to 0.005) | -0.009 (-0.02 to 0.005) | -0.01 (-0.02 to 0.002) |
| Secondary or more | 10,045 | -0.003 (-0.01 to 0.009) | -0.002 (-0.01 to 0.01) | -0.005 (-0.01 to 0.004) |
| P for interaction |  | 0.62 | 0.59 | 0.61 |
| **WC z-score** |  |  |  |  |
| Primary or less | 6,325 | -0.01 (-0.03 to 0.0007) | -0.01 (-0.03 to 0.002) | -0.02 (-0.03 to -0.004)^*^ |
| Secondary or more | 10,045 | -0.001 (-0.01 to 0.01) | -0.0007 (-0.01 to 0.01) | -0.007 (-0.02 to 0.003) |
| P for interaction |  | 0.07 | 0.09 | 0.15 |
| **HC z-score** |  |  |  |  |
| Primary or less | 6,325 | -0.01 (-0.03 to 0.003) | -0.01 (-0.03 to 0.003) | -0.002 (-0.01 to 0.01) |
| Secondary or more | 10,045 | -0.001 (-0.01 to 0.01) | -0.0008 (-0.01 to 0.01) | 0.0004 (-0.01 to 0.01) |
| P for interaction |  | 0.29 | 0.26 | 0.82 |
| **WHR z-score** |  |  |  |  |
| Primary or less | 6,325 | -0.01 (-0.03 to 0.005) | -0.009 (-0.02 to 0.006) | -0.02 (-0.04 to -0.01)^***^ |
| Secondary or more | 10,045 | -0.001 (-0.01 to 0.01) | -0.001 (-0.01 to 0.01) | -0.01 (-0.02 to -0.002)^*^ |
| P for interaction |  | 0.09 | 0.14 | 0.06 |
| **WHtR z-score** |  |  |  |  |
| Primary or less | 6,325 | -0.01 (-0.03 to 0.004) | -0.01 (-0.02 to 0.005) | -0.02 (-0.03 to -0.006)^**^ |
| Secondary or more | 10,045 | -0.001 (-0.01 to 0.01) | -0.001 (-0.01 to 0.01) | -0.01 (-0.02 to -0.0001)^*^ |
| P for interaction |  | 0.16 | 0.16 | 0.05 |
| **LAP z-score** |  |  |  |  |
| Primary or less | 6,268 | -0.01 (-0.03 to 0.002) | -0.01 (-0.03 to 0.002) | -0.01 (-0.02 to 0.0006) |
| Secondary or more | 9,927 | 0.001 (-0.01 to 0.01) | 0.002 (-0.009 to 0.01) | 0.00001 (-0.01 to 0.01) |
| P for interaction |  | 0.15 | 0.15 | 0.12 |
| **ABSI z-score** |  |  |  |  |
| Primary or less | 6,325 | -0.007 (-0.02 to 0.008) | -0.005 (-0.02 to 0.01) | -0.02 (-0.03 to -0.003)^*^ |
| Secondary or more | 10,045 | 0.003 (-0.009 to 0.02) | 0.003 (-0.01 to 0.01) | -0.007 (-0.02 to 0.003) |
| P for interaction |  | 0.03 | 0.05 | 0.03 |
| **VAI z-score** |  |  |  |  |
| Primary or less | 6,325 | -0.006 (-0.02 to 0.008) | -0.006 (-0.02 to 0.009) | -0.006 (-0.02 to 0.006) |
| Secondary or more | 10,045 | 0.003 (-0.008 to 0.01) | 0.003 (-0.008 to 0.01) | 0.003 (-0.006 to 0.01) |
| P for interaction |  | 0.47 | 0.50 | 0.30 |
| **CVAI z-score** |  |  |  |  |
| Primary or less | 6,325 | -0.02 (-0.03 to -0.001)^*^ | -0.02 (-0.03 to -0.001)^*^ | -0.02 (-0.03 to -0.003)^*^ |
| Secondary or more | 10,045 | 0.003 (-0.008 to 0.01) | 0.004 (-0.008 to 0.02) | 0.002 (-0.008 to 0.01) |
| P for interaction |  | 0.009 | 0.009 | 0.002 |
| **BRI z-score** |  |  |  |  |
| Primary or less | 6,325 | -0.01 (-0.02 to 0.004) | -0.01 (-0.02 to 0.005) | -0.02 (-0.03 to -0.006)^**^ |
| Secondary or more | 10,045 | -0.001 (-0.01 to 0.01) | -0.001 (-0.01 to 0.01) | -0.01 (-0.02 to -0.00006)^*^ |
| P for interaction |  | 0.15 | 0.16 | 0.06 |
| **Conicity index z-score** |  |  |  |  |
| Primary or less | 6,325 | 0.005 (-0.01 to 0.02) | 0.007 (-0.01 to 0.02) | -0.009 (-0.02 to 0.004) |
| Secondary or more | 10,045 | 0.005 (-0.008 to 0.02) | 0.003 (-0.01 to 0.02) | -0.005 (-0.02 to 0.006) |
| P for interaction |  | 0.37 | 0.41 | 0.03 |
| **BAI z-score** |  |  |  |  |
| Primary or less | 6,325 | -0.004 (-0.02 to 0.01) | -0.004 (-0.02 to 0.01) | -0.007 (-0.02 to 0.007) |
| Secondary or more | 10,045 | -0.001 (-0.02 to 0.01) | -0.002 (-0.02 to 0.01) | -0.005 (-0.02 to 0.006) |
| P for interaction |  | 0.87 | 0.76 | 0.22 |
| **CMI z-score** |  |  |  |  |
| Primary or less | 6,325 | -0.005 (-0.02 to 0.01) | -0.005 (-0.02 to 0.01) | -0.006 (-0.02 to 0.007) |
| Secondary or more | 10,045 | 0.004 (-0.007 to 0.01) | 0.004 (-0.007 to 0.01) | 0.003 (-0.005 to 0.01) |
| P for interaction |  | 0.41 | 0.46 | 0.36 |
| **BSA z-score** |  |  |  |  |
| Primary or less | 6,325 | -0.02 (-0.03 to -0.0008)^*^ | -0.02 (-0.03 to -0.0007)^*^ | -0.004 (-0.02 to 0.01) |
| Secondary or more | 10,045 | -0.003 (-0.02 to 0.01) | -0.001 (-0.01 to 0.01) | 0.001 (-0.009 to 0.01) |
| P for interaction |  | 0.25 | 0.30 | 0.46 |
| **WHHR z-score** |  |  |  |  |
| Primary or less | 6,325 | -0.005 (-0.02 to 0.01) | -0.004 (-0.02 to 0.01) | -0.03 (-0.04 to -0.02)^***^ |
| Secondary or more | 10,045 | -0.001 (-0.01 to 0.01) | -0.002 (-0.01 to 0.01) | -0.01 (-0.02 to -0.004)^**^ |
| P for interaction |  | 0.29 | 0.33 | 0.01 |
| **PFM z-score** |  |  |  |  |
| Primary or less | 6,325 | -0.01 (-0.03 to 0.002) | -0.01 (-0.03 to 0.002) | -0.01 (-0.03 to 0.002) |
| Secondary or more | 10,045 | -0.0007 (-0.01 to 0.01) | 0.0006 (-0.01 to 0.01) | -0.004 (-0.01 to 0.007) |
| P for interaction |  | 0.63 | 0.52 | 0.28 |
| **PLM z-score** |  |  |  |  |
| Primary or less | 6,325 | -0.03 (-0.05 to 0.001) | -0.03 (-0.05 to 0.001) | -0.004 (-0.03 to 0.02) |
| Secondary or more | 10,045 | -0.007 (-0.03 to 0.01) | -0.004 (-0.02 to 0.02) | 0.004 (-0.01 to 0.02) |
| P for interaction |  | 0.32 | 0.43 | 0.21 |
| **PPF z-score** |  |  |  |  |
| Primary or less | 6,325 | -0.02 (-0.06 to 0.01) | -0.02 (-0.06 to 0.01) | -0.03 (-0.06 to -0.004)^*^ |
| Secondary or more | 10,045 | 0.004 (-0.02 to 0.03) | 0.006 (-0.02 to 0.03) | -0.01 (-0.03 to 0.01) |
| P for interaction |  | 0.93 | 0.88 | 0.08 |
| **CUN-BAE z-score** |  |  |  |  |
| Primary or less | 6,325 | -0.02 (-0.04 to 0.008) | -0.02 (-0.04 to 0.008) | -0.02 (-0.04 to 0.003) |
| Secondary or more | 10,045 | -0.004 (-0.02 to 0.01) | -0.003 (-0.02 to 0.02) | -0.008 (-0.02 to 0.007) |
| P for interaction |  | 0.95 | 0.80 | 0.11 |

ABSI: a body shape index; BAI: body adiposity index; BMI: body mass index; BRI: body roundness index; BSA: body surface area; CI: confidence interval; CMI: cardiometabolic index; CUN-BAE: Clínica Universidad de Navarra-Body Adiposity Estimator; CVAI: Chinese visceral adiposity index; DWRT: Delayed Word Recall Test; HC, hip circumference; LAP: lipid accumulation product; PFM: predicted fat mass; PLM: predicted lean mass; PPF: predicated percent fat; Ref, reference; VAI: visceral adiposity index; WC: waist circumference; WHHR: waist-to-hip-to-height ratio; WHR: waist-to-hip ratio; WHtR: waist-to-height ratio

^a^: Adjusted for sex and age

^b^: Additionally adjusted for occupation, personal income, physical activity, drinking, smoking and self-rated health

^c^: Additionally adjusted for baseline DWRT score

^*^P <0.05, ^**^P <0.01, ^***^P <0.001

Underweight: BMI <18.5 kg/m^2^; Normal: 18.5 kg/m^2^ ≤ BMI <25 kg/m^2^; Overweight: 25 kg/m^2^ ≤ BMI < 27.5 kg/m^2^; Obese: BMI ≥ 27.5 kg/m^2^; High BMI: BMI ≥ 25 kg/m^2^

Normal WC: <90 cm for men, <80 cm for women; High WC: ≥90 cm for men, ≥80 cm for women

Normal WHR: <0.9 for men, <0.8 for women; High WHR: ≥0.9 for men, ≥0.8 for women

Normal WHtR: <0.5; High WHR: ≥0.5

Table S10. Associations of baseline obesity indicators with mean annual change rate of memory function.

|  | *N* | Adjusted mean differences β (95% CI) in mean annual change rate of DWRT score | | |
| --- | --- | --- | --- | --- |
|  |  | Model 1^a^ | Model 2^b^ | Model 3^c^ |
| Weight, kg | 16,271 | -0.02 (-0.05 to 0.002) | -0.02 (-0.05 to 0.002) | -0.02 (-0.04 to 0.002) |
| BMI, kg/m^2^ | 16,271 | -0.04 (-0.11 to 0.03) | -0.05 (-0.11 to 0.02) | -0.07 (-0.13 to -0.01)^*^ |
| WC, cm | 16,271 | -0.03 (-0.05 to 0.0005) | -0.03 (-0.06 to -0.003)^*^ | -0.04 (-0.07 to -0.02)^***^ |
| HC, cm | 16,271 | -0.03 (-0.07 to 0.002) | -0.03 (-0.07 to 0.0008) | -0.02 (-0.05 to 0.01) |
| WHR | 16,271 | -2.33 (-5.92 to 1.25) | -2.98 (-6.59 to 0.63) | -7.59 (-10.62 to -4.55)^***^ |
| WHtR | 16,271 | -2.88 (-6.95 to 1.18) | -3.82 (-7.93 to 0.29) | -7.51 (-10.96 to -4.06)^***^ |
| LAP | 16,096 | -0.003 (-0.01 to 0.004) | -0.004 (-0.01 to 0.003) | -0.005 (-0.01 to 0.001) |
| ABSI | 16,271 | -26.21 (-75.68 to 23.27) | -34.73 (-84.77 to 15.31) | -79.57 (-121.64 to -37.50)^***^ |
| VAI | 16,271 | 0.02 (-0.10 to 0.14) | 0.02 (-0.11 to 0.14) | 0.006 (-0.10 to 0.11) |
| CVAI | 16,271 | -0.003 (-0.009 to 0.003) | -0.004 (-0.009 to 0.002) | -0.005 (-0.01 to 0.0001) |
| BRI | 16,271 | -0.15 (-0.36 to 0.06) | -0.20 (-0.41 to 0.02) | -0.40 (-0.58 to -0.22)^***^ |
| Conicity index | 16,271 | 7.72 (-9.72 to 25.15) | 5.06 (-12.56 to 22.68) | -11.44 (-26.26 to 3.38) |
| BAI | 16,271 | -0.01 (-0.08 to 0.05) | -0.03 (-0.09 to 0.04) | -0.04 (-0.10 to 0.01) |
| CMI | 16,271 | 0.10 (-0.27 to 0.48) | 0.08 (-0.29 to 0.46) | 0.04 (-0.27 to 0.36) |
| BSA | 16,271 | -2.05 (-3.84 to -0.26)^*^ | -1.91 (-3.70 to -0.11)^*^ | -1.04 (-2.55 to 0.47) |
| WHHR | 16,271 | -95.81 (-599.82 to 408.19) | -227.96 (-737.35 to 281.43) | -1042.08 (-1470.51 to -613.65)^***^ |
| PFM | 16,271 | -0.03 (-0.07 to 0.01) | -0.03 (-0.08 to 0.008) | -0.04 (-0.08 to -0.008)^*^ |
| PLM | 16,271 | -0.06 (-0.11 to -0.004)^*^ | -0.05 (-0.11 to 0.001) | -0.02 (-0.07 to 0.03) |
| PPF | 16,271 | -0.03 (-0.10 to 0.04) | -0.04 (-0.11 to 0.03) | -0.09 (-0.15 to -0.03)^**^ |
| CUN-BAE | 16,271 | -0.03 (-0.09 to 0.02) | -0.04 (-0.09 to 0.02) | -0.06 (-0.10 to -0.01)^*^ |
| Weight z-score | 16,271 | -0.22 (-0.46 to 0.02) | -0.22 (-0.46 to 0.02) | -0.19 (-0.39 to 0.02) |
| BMI z-score | 16,271 | -0.13 (-0.35 to 0.10) | -0.15 (-0.38 to 0.07) | -0.24 (-0.43 to -0.05)^*^ |
| WC z-score | 16,271 | -0.23 (-0.46 to 0.005) | -0.26 (-0.50 to -0.03)^*^ | -0.40 (-0.59 to -0.20)^***^ |
| HC z-score | 16,271 | -0.21 (-0.43 to 0.01) | -0.22 (-0.44 to 0.005) | -0.12 (-0.31 to 0.07) |
| WHR z-score | 16,271 | -0.16 (-0.40 to 0.08) | -0.20 (-0.45 to 0.04) | -0.51 (-0.72 to -0.31)^***^ |
| WHtR z-score | 16,271 | -0.16 (-0.40 to 0.07) | -0.22 (-0.45 to 0.02) | -0.43 (-0.63 to -0.23)^***^ |
| LAP z-score | 16,096 | -0.10 (-0.33 to 0.13) | -0.12 (-0.36 to 0.11) | -0.17 (-0.36 to 0.03) |
| ABSI z-score | 16,271 | -0.13 (-0.37 to 0.11) | -0.17 (-0.42 to 0.08) | -0.39 (-0.60 to -0.18)^***^ |
| VAI z-score | 16,271 | 0.04 (-0.18 to 0.27) | 0.03 (-0.20 to 0.25) | 0.01 (-0.18 to 0.20) |
| CVAI z-score | 16,271 | -0.12 (-0.36 to 0.11) | -0.15 (-0.39 to 0.08) | -0.19 (-0.39 to 0.005) |
| BRI z-score | 16,271 | -0.16 (-0.40 to 0.07) | -0.22 (-0.45 to 0.02) | -0.44 (-0.64 to -0.24)^***^ |
| Conicity index z-score | 16,271 | 0.11 (-0.14 to 0.36) | 0.07 (-0.18 to 0.32) | -0.16 (-0.37 to 0.05) |
| BAI z-score | 16,271 | -0.06 (-0.32 to 0.21) | -0.11 (-0.38 to 0.15) | -0.18 (-0.41 to 0.04) |
| CMI z-score | 16,271 | 0.06 (-0.16 to 0.29) | 0.05 (-0.17 to 0.27) | 0.02 (-0.16 to 0.21) |
| BSA z-score | 16,271 | -0.30 (-0.56 to -0.04)^*^ | -0.28 (-0.54 to -0.02)^*^ | -0.15 (-0.37 to 0.07) |
| WHHR z-score | 16,271 | -0.05 (-0.28 to 0.19) | -0.11 (-0.35 to 0.13) | -0.49 (-0.69 to -0.29)^***^ |
| PFM z-score | 16,271 | -0.20 (-0.46 to 0.07) | -0.21 (-0.48 to 0.05) | -0.27 (-0.49 to -0.05)^*^ |
| PLM z-score | 16,271 | -0.44 (-0.85 to -0.03)^*^ | -0.40 (-0.82 to 0.009) | -0.15 (-0.50 to 0.20) |
| PPF z-score | 16,271 | -0.21 (-0.73 to 0.31) | -0.28 (-0.80 to 0.24) | -0.63 (-1.07 to -0.19)^**^ |
| CUN-BAE z-score | 16,271 | -0.22 (-0.59 to 0.15) | -0.26 (-0.63 to 0.11) | -0.38 (-0.69 to -0.07)^*^ |
| BMI groups |  |  |  |  |
| Underweight | 666 | 1.53 (0.40 to 2.65)^**^ | 1.44 (0.31 to 2.57)^*^ | 0.72 (-0.23 to 1.67) |
| Normal | 10,123 | Ref. (0) | Ref. (0) | Ref. (0) |
| Overweight | 3,456 | 0.29 (-0.27 to 0.84) | 0.24 (-0.32 to 0.79) | -0.003 (-0.47 to 0.46) |
| Obese | 2,026 | -0.08 (-0.77 to 0.60) | -0.18 (-0.86 to 0.51) | -0.45 (-1.03 to 0.13) |
| WC groups |  |  |  |  |
| Normal | 10,908 | Ref. (0) | Ref. (0) | Ref. (0) |
| High | 5,363 | -0.71 (-1.19 to -0.23)^**^ | -0.81 (-1.30 to -0.33)^**^ | -0.98 (-1.39 to -0.58)^***^ |
| BMI & WC groups |  |  |  |  |
| Non-obese | 9,312 | Ref. (0) | Ref. (0) | Ref. (0) |
| High BMI | 1,596 | 0.89 (0.12 to 1.65)^*^ | 0.94 (0.17 to 1.70)^*^ | 0.75 (-0.11 to 1.39)^*^ |
| High WC | 1,477 | -0.91 (-1.72 to -0.11)^*^ | -0.96 (-1.76 to -0.15)^*^ | -1.04 (1.72 to -0.37)^**^ |
| High BMI & WC | 3,886 | -0.47 (-1.02 to 0.07) | -0.59 (-1.13 to -0.04)^*^ | -0.83 (-1.28 to -0.37)^***^ |
| WHR groups |  |  |  |  |
| Normal | 4,721 | Ref. (0) | Ref. (0) | Ref. (0) |
| High | 11,550 | -0.06 (-0.58 to 0.45) | -0.14 (-0.65 to 0.38) | -0.63 (-1.06 to -0.19)^**^ |
| WHtR groups |  |  |  |  |
| Normal | 8,118 | Ref. (0) | Ref. (0) | Ref. (0) |
| High | 8,153 | -0.30 (-0.75 to 0.15) | -0.41 (-0.86 to 0.04) | -0.78 (-1.16 to -0.40)^***^ |

ABSI: a body shape index; BAI: body adiposity index; BMI: body mass index; BRI: body roundness index; BSA: body surface area; CI: confidence interval; CMI: cardiometabolic index; CUN-BAE: Clínica Universidad de Navarra-Body Adiposity Estimator; CVAI: Chinese visceral adiposity index; DWRT: Delayed Word Recall Test; HC, hip circumference; LAP: lipid accumulation product; PFM: predicted fat mass; PLM: predicted lean mass; PPF: predicated percent fat; Ref, reference; VAI: visceral adiposity index; WC: waist circumference; WHHR: waist-to-hip-to-height ratio; WHR: waist-to-hip ratio; WHtR: waist-to-height ratio

^a^: Adjusted for sex and age

^b^: Additionally adjusted for education, occupation, personal income, physical activity, drinking, smoking and self-rated health

^c^: Additionally adjusted for baseline DWRT score

^*^P <0.05, ^**^P <0.01, ^***^P <0.001

Underweight: BMI <18.5 kg/m^2^; Normal: 18.5 kg/m^2^ ≤ BMI <25 kg/m^2^; Overweight: 25 kg/m^2^ ≤ BMI < 27.5 kg/m^2^; Obese: BMI ≥ 27.5 kg/m^2^; High BMI: BMI ≥ 25 kg/m^2^

Normal WC: <90 cm for men, <80 cm for women; High WC: ≥90 cm for men, ≥80 cm for women

Normal WHR: <0.9 for men, <0.8 for women; High WHR: ≥0.9 for men, ≥0.8 for women

Normal WHtR: <0.5; High WHR: ≥0.5

Table S11. Associations of baseline obesity indicators with mean annual change rate of memory function by education.

|  | *N* | Adjusted mean differences β (95% CI) in mean annual change rate of DWRT score | | |
| --- | --- | --- | --- | --- |
|  |  | Model 1^a^ | Model 2^b^ | Model 3^c^ |
| **Weight z-score** |  |  |  |  |
| Primary or less | 6,264 | -0.54 (-0.99 to -0.09)^*^ | -0.55 (-1.00 to -0.09)^*^ | -0.35 (-0.74 to 0.04) |
| Secondary or more | 10,007 | -0.06 (-0.33 to 0.21) | -0.03 (-0.30 to 0.24) | -0.05 (-0.27 to 0.17) |
| P for interaction |  | 0.17 | 0.18 | 0.70 |
| **BMI z-score** |  |  |  |  |
| Primary or less | 6,264 | -0.37 (-0.79 to 0.04) | -0.38 (-0.79 to 0.03) | -0.42 (-0.77 to -0.07)^*^ |
| Secondary or more | 10,007 | -0.01 (-0.27 to 0.24) | 0.002 (-0.26 to 0.26) | -0.09 (-0.30 to 0.12) |
| P for interaction |  | 0.16 | 0.16 | 0.13 |
| **WC z-score** |  |  |  |  |
| Primary or less | 6,264 | -0.60 (-1.03 to -0.17)^**^ | -0.57 (-1.01 to -0.14)^*^ | -0.65 (-1.03 to -0.28)^**^ |
| Secondary or more | 10,007 | -0.06 (-0.33 to 0.20) | -0.05 (-0.32 to 0.21) | -0.19 (-0.40 to 0.03) |
| P for interaction |  | 0.02 | 0.02 | 0.03 |
| **HC z-score** |  |  |  |  |
| Primary or less | 6,264 | -0.53 (-0.94 to -0.12)^*^ | -0.53 (-0.94 to -0.11)^*^ | -0.25 (-0.60 to 0.11) |
| Secondary or more | 10,007 | -0.04 (-0.29 to 0.22) | -0.02 (-0.27 to 0.23) | 0.0002 (-0.21 to 0.21) |
| P for interaction |  | 0.04 | 0.03 | 0.14 |
| **WHR z-score** |  |  |  |  |
| Primary or less | 6,264 | -0.41 (-0.86 to 0.04) | -0.37 (-0.82 to 0.09) | -0.78 (-1.16 to -0.39)^***^ |
| Secondary or more | 10,007 | -0.08 (-0.36 to 0.20) | -0.08 (-0.35 to 0.20) | -0.31 (-0.54 to -0.09)^**^ |
| P for interaction |  | 0.11 | 0.15 | 0.07 |
| **WHtR z-score** |  |  |  |  |
| Primary or less | 6,264 | -0.50 (-0.92 to -0.07)^*^ | -0.47 (-0.89 to -0.04)^*^ | -0.70 (-1.07 to -0.33)^***^ |
| Secondary or more | 10,007 | -0.04 (-0.30 to 0.23) | -0.03 (-0.30 to 0.23) | -0.21 (-0.43 to 0.01) |
| P for interaction |  | 0.02 | 0.02 | 0.004 |
| **LAP z-score** |  |  |  |  |
| Primary or less | 6,207 | -0.28 (-0.72 to 0.15) | -0.29 (-0.72 to 0.15) | -0.27 (-0.64 to 0.10) |
| Secondary or more | 9,889 | -0.04 (-0.30 to 0.22) | -0.02 (-0.29 to 0.24) | -0.07 (-0.29 to 0.14) |
| P for interaction |  | 0.30 | 0.31 | 0.30 |
| **ABSI z-score** |  |  |  |  |
| Primary or less | 6,264 | -0.37 (-0.82 to 0.07) | -0.32 (-0.77 to 0.14) | -0.58 (-0.97 to -0.20)^**^ |
| Secondary or more | 10,007 | -0.04 (-0.32 to 0.24) | -0.05 (-0.33 to 0.23) | -0.23 (-0.46 to -0.004)^*^ |
| P for interaction |  | 0.04 | 0.049 | 0.03 |
| **VAI z-score** |  |  |  |  |
| Primary or less | 6,264 | -0.004 (-0.43 to 0.42) | -0.02 (-0.44 to 0.41) | -0.02 (-0.38 to 0.35) |
| Secondary or more | 10,007 | 0.04 (-0.21 to 0.29) | 0.04 (-0.21 to 0.29) | 0.03 (-0.18 to 0.23) |
| P for interaction |  | 0.93 | 0.98 | 0.87 |
| **CVAI z-score** |  |  |  |  |
| Primary or less | 6,264 | -0.52 (-0.97 to -0.06)^*^ | -0.52 (-0.98 to -0.06)^*^ | -0.50 (-0.89 to -0.11)^*^ |
| Secondary or more | 10,007 | 0.05 (-0.21 to 0.31) | 0.06 (-0.20 to 0.32) | 0.01 (-0.20 to 0.23) |
| P for interaction |  | 0.004 | 0.004 | 0.001 |
| **BRI z-score** |  |  |  |  |
| Primary or less | 6,264 | -0.48 (-0.90 to -0.06)^*^ | -0.46 (-0.88 to -0.04)^*^ | -0.70 (-1.06 to -0.34)^***^ |
| Secondary or more | 10,007 | -0.02 (-0.30 to 0.25) | -0.02 (-0.30 to 0.25) | -0.21 (-0.44 to 0.01) |
| P for interaction |  | 0.02 | 0.02 | 0.005 |
| **Conicity index z-score** |  |  |  |  |
| Primary or less | 6,264 | 0.12 (-0.33 to 0.57) | 0.17 (-0.28 to 0.63) | -0.23 (-0.62 to 0.16) |
| Secondary or more | 10,007 | 0.06 (-0.22 to 0.34) | 0.02 (-0.26 to 0.31) | -0.12 (-0.35 to 0.11) |
| P for interaction |  | 0.47 | 0.49 | 0.08 |
| **BAI z-score** |  |  |  |  |
| Primary or less | 6,264 | -0.31 (-0.79 to 0.16) | -0.31 (-0.78 to 0.17) | -0.37 (-0.77 to 0.04) |
| Secondary or more | 10,007 | 0.05 (-0.26 to 0.36) | 0.04 (-0.27 to 0.35) | -0.03 (-0.28 to 0.22) |
| P for interaction |  | 0.11 | 0.09 | 0.009 |
| **CMI z-score** |  |  |  |  |
| Primary or less | 6,264 | -0.0005 (-0.44 to 0.44) | -0.01 (-0.45 to 0.42) | -0.04 (-0.41 to 0.34) |
| Secondary or more | 10,007 | 0.07 (-0.18 to 0.31) | 0.07 (-0.17 to 0.32) | 0.06 (-0.14 to 0.26) |
| P for interaction |  | 0.88 | 0.94 | 0.92 |
| **BSA z-score** |  |  |  |  |
| Primary or less | 6,264 | -0.64 (-1.14 to -0.14)^*^ | -0.64 (-1.14 to -0.14)^*^ | -0.27 (-0.70 to 0.16) |
| Secondary or more | 10,007 | -0.10 (-0.39 to 0.19) | -0.07 (-0.36 to 0.22) | -0.03 (-0.27 to 0.21) |
| P for interaction |  | 0.24 | 0.27 | 0.70 |
| **WHHR z-score** |  |  |  |  |
| Primary or less | 6,264 | -0.22 (-0.66 to 0.22) | -0.18 (-0.62 to 0.26) | -0.77 (-1.15 to -0.39)^***^ |
| Secondary or more | 10,007 | -0.02 (-0.30 to 0.25) | -0.04 (-0.31 to 0.24) | -0.30 (-0.52 to -0.07)^**^ |
| P for interaction |  | 0.15 | 0.19 | 0.008 |
| **PFM z-score** |  |  |  |  |
| Primary or less | 6,264 | -0.57 (-1.06 to -0.08)^*^ | -0.58 (-1.07 to -0.09)^*^ | -0.51 (-0.93 to -0.09)^*^ |
| Secondary or more | 10,007 | -0.004 (-0.30 to 0.29) | 0.02 (-0.28 to 0.32) | -0.08 (-0.33 to 0.16) |
| P for interaction |  | 0.08 | 0.06 | 0.02 |
| **PLM z-score** |  |  |  |  |
| Primary or less | 6,264 | -0.88 (-1.68 to -0.08)^*^ | -0.89 (-1.70 to -0.09)^*^ | -0.28 (-0.97 to 0.41) |
| Secondary or more | 10,007 | -0.20 (-0.65 to 0.25) | -0.14 (-0.59 to 0.31) | -0.007 (-0.37 to 0.36) |
| P for interaction |  | 0.71 | 0.82 | 0.12 |
| **PPF z-score** |  |  |  |  |
| Primary or less | 6,264 | -1.04 (-2.03 to -0.05)^*^ | -1.03 (-2.02 to -0.04)^*^ | -1.31 (-2.16 to -0.47)^**^ |
| Secondary or more | 10,007 | 0.16 (-0.42 to 0.74) | 0.19 (-0.39 to 0.77) | -0.15 (-0.63 to 0.32) |
| P for interaction |  | 0.18 | 0.13 | 0.003 |
| **CUN-BAE z-score** |  |  |  |  |
| Primary or less | 6,264 | -0.65 (-1.35 to 0.04) | -0.66 (-1.36 to 0.04) | -0.70 (-1.30 to -0.11)^*^ |
| Secondary or more | 10,007 | -0.03 (-0.45 to 0.38) | -0.008 (-0.42 to 0.41) | -0.14 (-0.48 to 0.20) |
| P for interaction |  | 0.14 | 0.11 | 0.004 |

ABSI: a body shape index; BAI: body adiposity index; BMI: body mass index; BRI: body roundness index; BSA: body surface area; CI: confidence interval; CMI: cardiometabolic index; CUN-BAE: Clínica Universidad de Navarra-Body Adiposity Estimator; CVAI: Chinese visceral adiposity index; DWRT: Delayed Word Recall Test; HC, hip circumference; LAP: lipid accumulation product; PFM: predicted fat mass; PLM: predicted lean mass; PPF: predicated percent fat; Ref, reference; VAI: visceral adiposity index; WC: waist circumference; WHHR: waist-to-hip-to-height ratio; WHR: waist-to-hip ratio; WHtR: waist-to-height ratio

^a^: Adjusted for sex and age

^b^: Additionally adjusted for occupation, personal income, physical activity, drinking, smoking and self-rated health

^c^: Additionally adjusted for baseline DWRT score

^*^P <0.05, ^**^P <0.01, ^***^P <0.001

Underweight: BMI <18.5 kg/m^2^; Normal: 18.5 kg/m^2^ ≤ BMI <25 kg/m^2^; Overweight: 25 kg/m^2^ ≤ BMI < 27.5 kg/m^2^; Obese: BMI ≥ 27.5 kg/m^2^; High BMI: BMI ≥ 25 kg/m^2^

Normal WC: <90 cm for men, <80 cm for women; High WC: ≥90 cm for men, ≥80 cm for women

Normal WHR: <0.9 for men, <0.8 for women; High WHR: ≥0.9 for men, ≥0.8 for women

Normal WHtR: <0.5; High WHR: ≥0.5


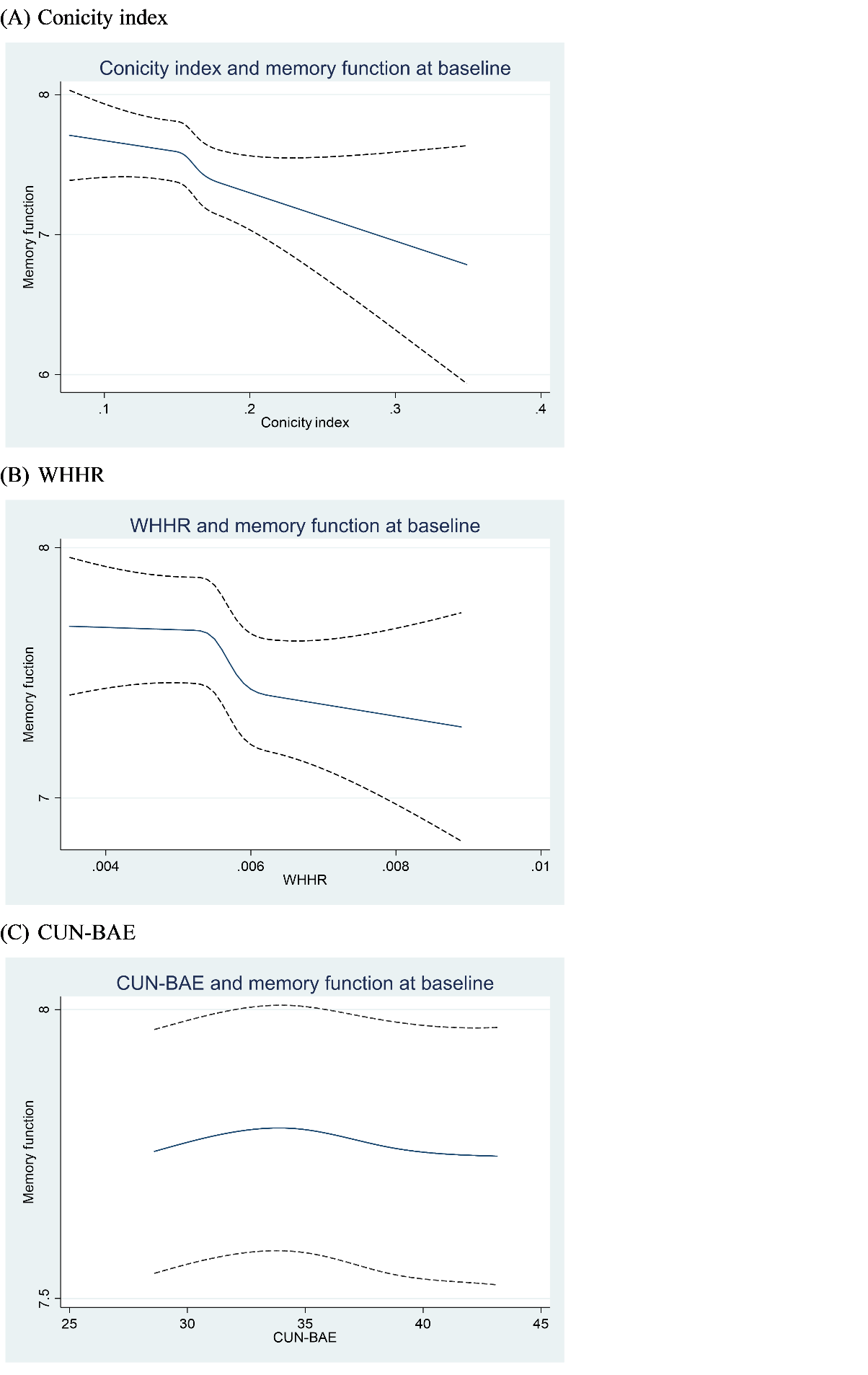


Figure S1. Non-linear associations of some obesity indicators with memory function at baseline: (A) Conicity index; (B) WHHR; (C) CUN-BAE

Note: CUN-BAE: Clínica Universidad de Navarra-Body Adiposity Estimator; WHHR: waist-to-hip-to-height ratio


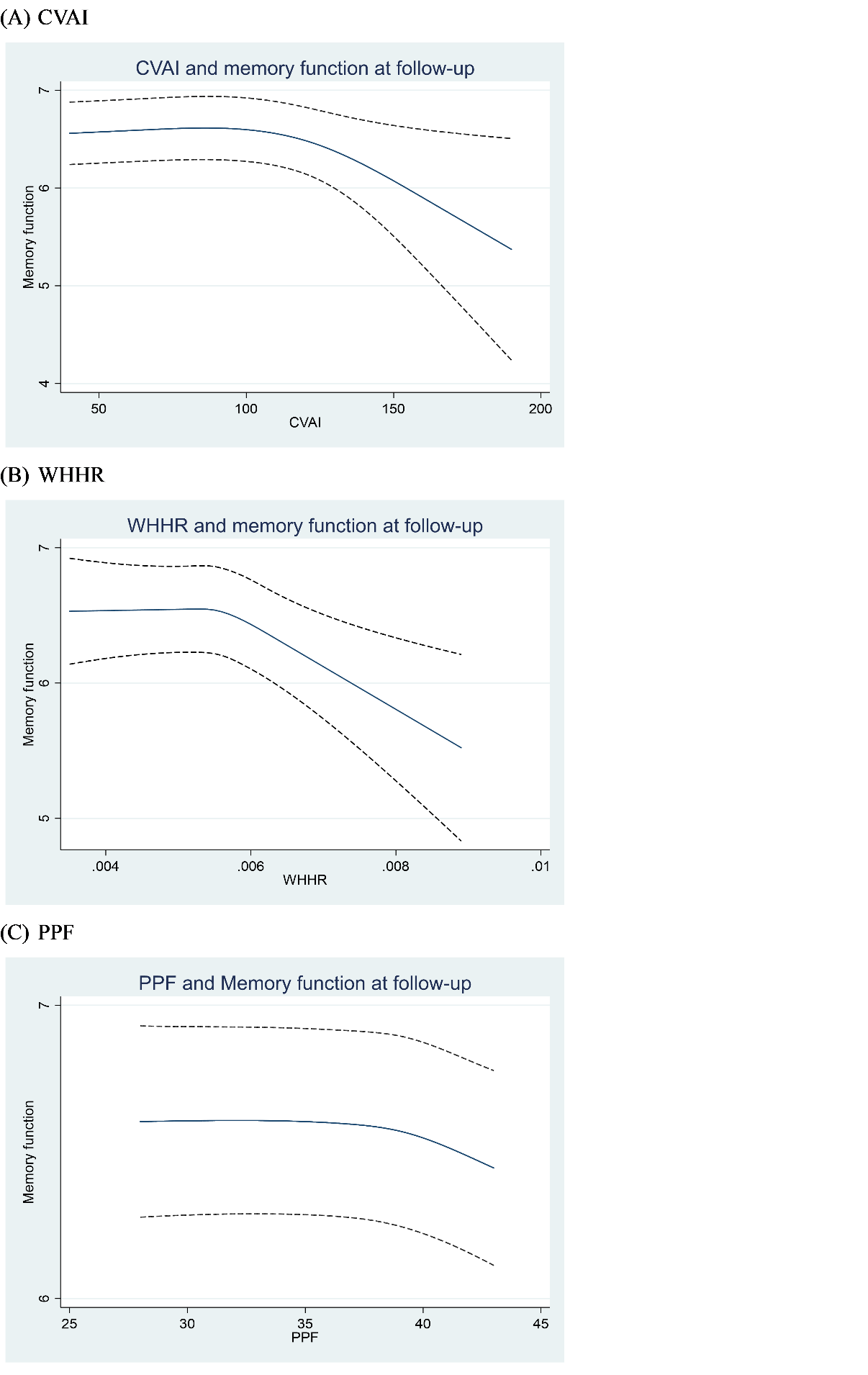


Figure S2. Non-linear associations of some obesity indicators with memory function at follow-up: (A) CVAI; (B) WHHR; (C) PPF

Note: CVAI: Chinese visceral adiposity index; PPF: predicated percent fat; WHHR: waist-to-hip-to-height ratio


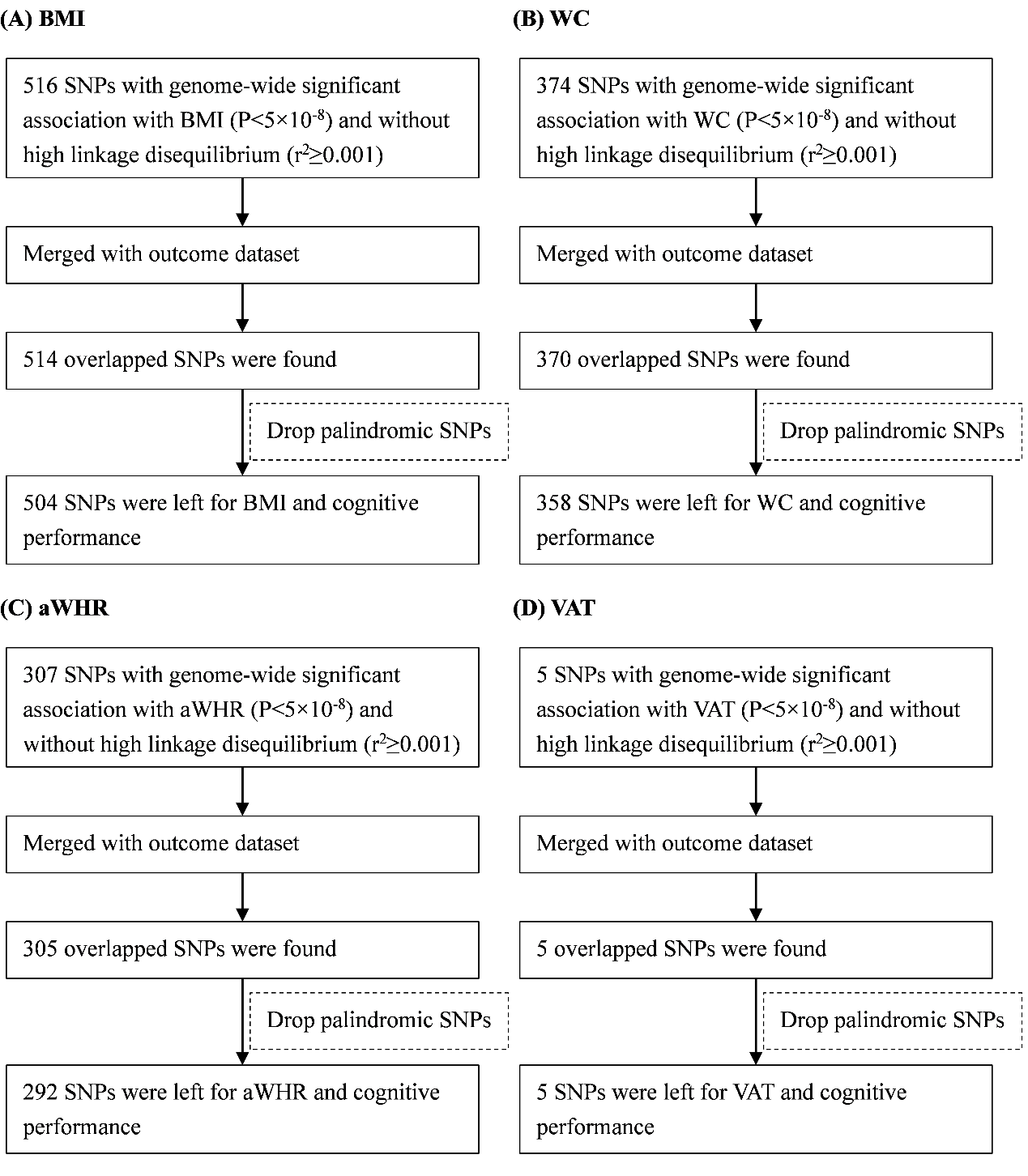


Figure S3. Flowchart showing selection of SNPs related to obesity used as instruments in analysis of effect on cognitive performance: (A) BMI; (B) WC; (C) aWHR; (D) VAT.

Note: aWHR: BMI-adjusted waist-to-hip ratio; BMI: body mass index; VAT: visceral adiposity tissue; WC: waist circumference
